# Knockdown of Hyaluronan synthase 2 suppresses liver fibrosis in mice via induction of transcriptomic changes similar to 4MU treatment

**DOI:** 10.1038/s41598-024-53089-x

**Published:** 2024-02-02

**Authors:** Noreen Halimani, Mikhail Nesterchuk, Alexandra A. Tsitrina, Marat Sabirov, Irina N. Andreichenko, Nataliya O. Dashenkova, Elizaveta Petrova, Alexey M. Kulikov, Timofei S. Zatsepin, Roman A. Romanov, Arsen S. Mikaelyan, Yuri V. Kotelevtsev

**Affiliations:** 1 Vladimir Zelman Center for Neurobiology and Brain Rehabilitation and Center of Life Sciences, Skolkovo Institute of Science and Technology, Moscow, 143025 Russia; 2 IKI-Ilse Katz Institute for Nanoscale Science & Technology, Nem Gurion University of the Negev, Beersheba, Israel; 3 Koltzov Institute of Developmental Biology of Russian Academy of Sciences, 26 Vavilov Street, Moscow, 119334 Russia; 4 AO Reproduction Head Centre of Agricultural Animals, Tsentralnaya Street, 3., Podolsk, Moscow Region, 142143 Russia; 5 Department of Chemistry, M.V. Lomonosov Moscow State University, Moscow, Russia; 6 Department of Molecular Neurosciences, Center for Brain Research, Medical University of Vienna, Vienna, Austria

**Keywords:** Cell biology, Drug discovery, Gastroenterology

## Abstract

Hepatic fibrosis remains a significant clinical challenge due to ineffective treatments. 4-methylumbelliferone (4MU), a hyaluronic acid (HA) synthesis inhibitor, has proven safe in phase one clinical trials. In this study, we aimed to ameliorate liver fibrosis by inhibiting HA synthesis. We compared two groups of mice with CCl_4_-induced fibrosis, treated with 4-methylumbelliferone (4MU) and hyaluronan synthase 2 (HAS2) targeting siRNA (siHAS2). The administration of 4MU and siHAS2 significantly reduced collagen and HA deposition, as well as biochemical markers of hepatic damage induced by repeated CCl_4_ injections. The transcriptomic analysis revealed converging pathways associated with downstream HA signalling. 4MU- and siHAS2-treated fibrotic livers shared 405 upregulated and 628 downregulated genes. These genes were associated with xenobiotic and cholesterol metabolism, mitosis, endoplasmic reticulum stress, RNA processing, and myeloid cell migration. The functional annotation of differentially expressed genes (DEGs) in siHAS2-treated mice revealed attenuation of extracellular matrix-associated pathways. In comparison, in the 4MU-treated group, DEGs were related to lipid and bile metabolism pathways and cell cycle. These findings confirm that HAS2 is an important pharmacological target for suppressing hepatic fibrosis using siRNA.

## Introduction

Hepatic fibrosis has a multifactorial aetiology and progresses rapidly towards severe and potentially life-threatening complications^1–4^. In fact, epidemiological data from phase 2b studies of simtuzumab (a humanised monoclonal antibody against LOXL2) in patients with advanced fibrosis revealed that one in every five patients develops cirrhosis within approximately 2.5 years^5^. Importantly, cirrhosis is recognised as a clinical risk factor for developing hepatocellular carcinoma^6,7^. Hepatic fibrosis is a pathological process arising from repeated liver damage; hence, current treatment options primarily aim to address the underlying cause of liver injury. However, therapies that solely target the reversal of fibrosis are limited by their side effects, rendering them unsuitable for long-term use (e.g., corticosteroids) or by a lack of proven efficacy (e.g., simtuzumab)^5,8^. Consequently, there are no treatment options that are both safe and effective, underscoring the need for expedited research into innovative therapeutic strategies.

Regardless of the underlying cause, the development of fibrosis involves inflammatory reactions. After liver injury, hepatocytes release damage-associated molecular patterns (DAMPs), which serve as danger signals that activate the innate immune system and trigger inflammation. This process is fueled by pro-inflammatory cytokines, such as interleukin-6 (IL-6), tumour necrosis factor-alpha (TNF-α), and other mediators released by tissue-resident macrophages (i.e., Kupffer cells) and infiltrating monocytes expressing high levels of Ly6C (LY6C^hi^)^9–11^. The non-resolving inflammation triggers the activation of quiescent hepatic stellate cells (qHSCs), which then transdifferentiate into α-SMA^hi^ myofibroblasts that produce copious amounts of extracellular matrix (ECM) components, starting with its major carbohydrate component, hyaluronic acid (HA) and associated proteins (e.g., fibrous collagen and elastin), adhesive proteins (e.g., laminin and fibronectin), resulting in the disruption of organ architecture and loss of function^12–15^. ECM deposition is the hallmark feature of fibrosis; thus, inhibiting the production of ECM could be a practical strategy for protection against or alleviating hepatic fibrosis.

HA is a vital component of ECM and is produced by three isoforms of hyaluronic acid synthase (HAS)^16–19^. Overproduction of HA is associated with organ fibrosis, cancer and inflammation. Multiple studies have shown that damaged tissues contain nearly 80 times more HA than healthy tissues^20,21^. The interaction of low molecular weight HA with its receptors CD44, RHAMM, and TLR causes local inflammation^22^, which has been linked to the development of chronic inflammatory disorders^23^. A study by Yang, Y. M. *et a*l. showed increased expression of HAS2 in advanced liver fibrosis caused by hepatitis B, hepatitis C, and NASH, confirmed in murine fibrosis models^24^. Furthermore, siRNA-mediated knockdown of HAS2 reduced fibrosis in CCl_4_-treated mice. Given the pivotal role of HA in the development and progression of several diseases, there are ongoing attempts to identify pharmacological interventions that can effectively inhibit HA synthesis^25–28^.

4-Methylumbelliferone (4MU; 7-hydroxy-4-methylcoumarin), sold under the trade name Hymecromone, is used to treat biliary spasms in Asia and Europe^29,30^. It is also a known inhibitor of HA synthesis. It has been suggested that 4MU inhibits HA production by depleting the cytosolic pool of the enzyme’s substrate—uridine diphosphate (UDP)-glucuronic acid^31–33^. Additionally, 4MU represses HAS2 and HAS3 mRNA expression^34,35^. In vivo, 4MU demonstrated therapeutic effects associated with the reduction of HA synthesis in liver, breast, and ovarian cancer and NASH^36–41^. To promote drug development and stimulate the repurposing of 4MU as an antifibrotic agent, it is crucial first to clarify the mechanism(s) of its action and elucidate its targets.

In our previous study, we demonstrated that 4MU inhibits HA and collagen deposition in the liver during CCl_4_-induced fibrosis^42^. Moreover, 4MU prevented the trans-differentiation of HSCs to myofibroblasts and altered the localisation pattern of myofibroblasts and macrophages. Total liver transcriptomic analysis of fibrotic mice treated with 4MU showed significant down-regulation of follistatin-like protein 1 (FSTL1), a cytokine downstream of TGF-β signal transduction. Furthermore, the analysis revealed other potential mechanisms of the antifibrotic effect of 4MU^42^. For instance, we observed a significant induction of Cyp3a11, a characteristic marker of the pregnane X receptor (PXR) activated by xenobiotics similar to 4MU. PXR activation suppresses inflammation^43^ through NF-kβ and AP-1 pathways, blunting CXCL2’s effect on macrophage chemotaxis^44^.

In this study, we compared the transcriptome signatures between mice with CCl_4_-induced fibrosis treated with 4MU and those that underwent knockdown of HAS2 using lipid nanoparticles containing small interfering RNA (siHAS2-LNP). This experimental design delineated the converging pathways regulated by HAS2 activity and 4MU treatment. We also describe several genes involved in cell adhesion and migration, which are integral processes in the progression of liver fibrosis.

## Results

### *4MU and siHAS2-LNP alleviate CCl*_*4*_*-induced liver fibrosis in mice*

**Figure 1:**
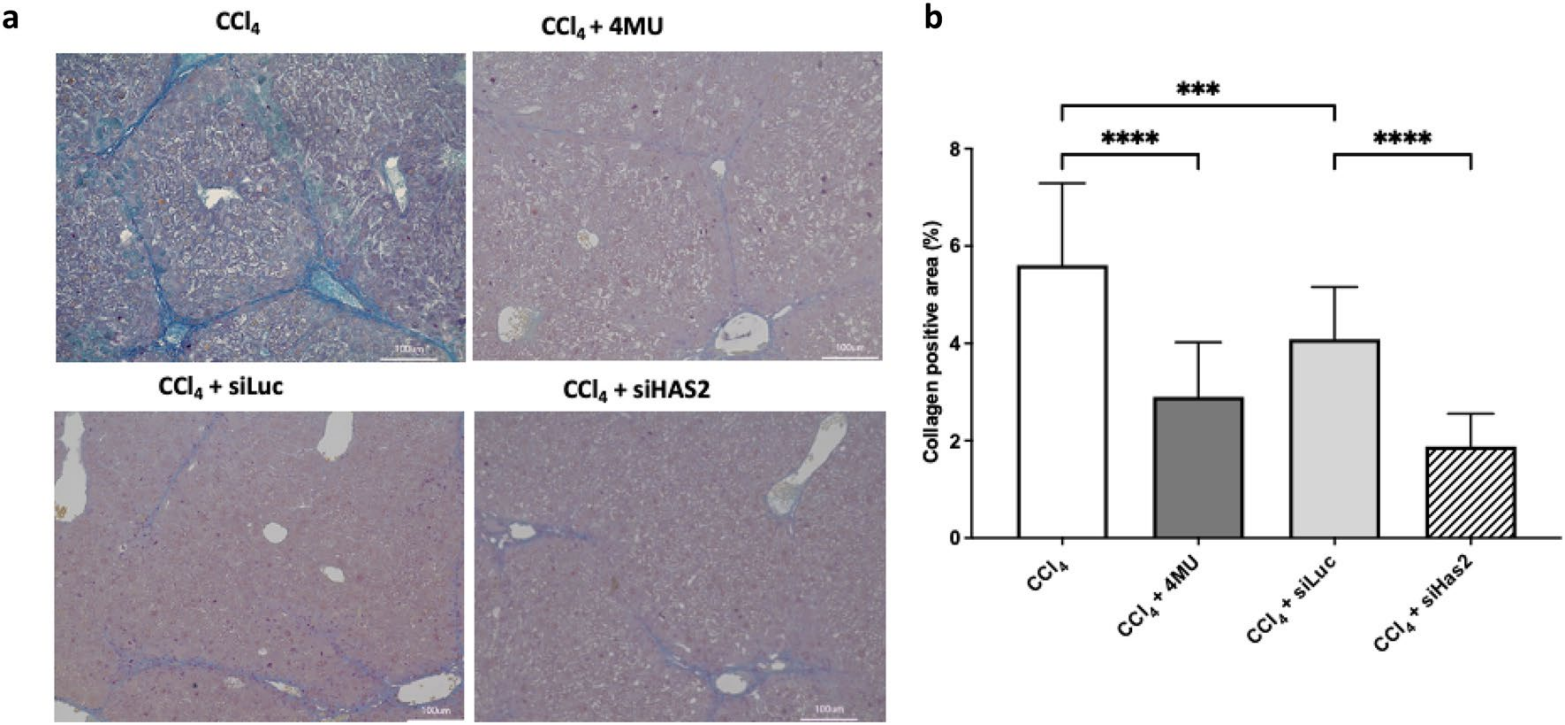
Collagen deposition in liver tissue of CCl_4_ -treated mice. (**a**) Masson’s trichrome staining of liver tissue. Scale bar: 100 µm. (**b**) Quantitative evaluation of collagen deposition. Data are presented as mean ± SD, n = 4. One-way ANOVA; **** p < 0,0001, ***p < 0,001.

The intraperitoneal administration of CCl_4_ for two weeks resulted in the dense deposition of collagen fibres around the portal triads. Treatment with both 4MU and lipid nanoparticle-encapsulated HAS2-targeting siRNA (siHAS2-LNP) significantly inhibited the deposition of collagen fibres. Interestingly, the administration of LNP-encapsulated luciferase-targeting siRNA (siLuc-LNP), which is a non-targeting control, also resulted in significantly inhibited collagen deposition, although not to the same extent as mice treated with 4MU or siHAS2 (Fig. 1a, b).

**Figure 2:**
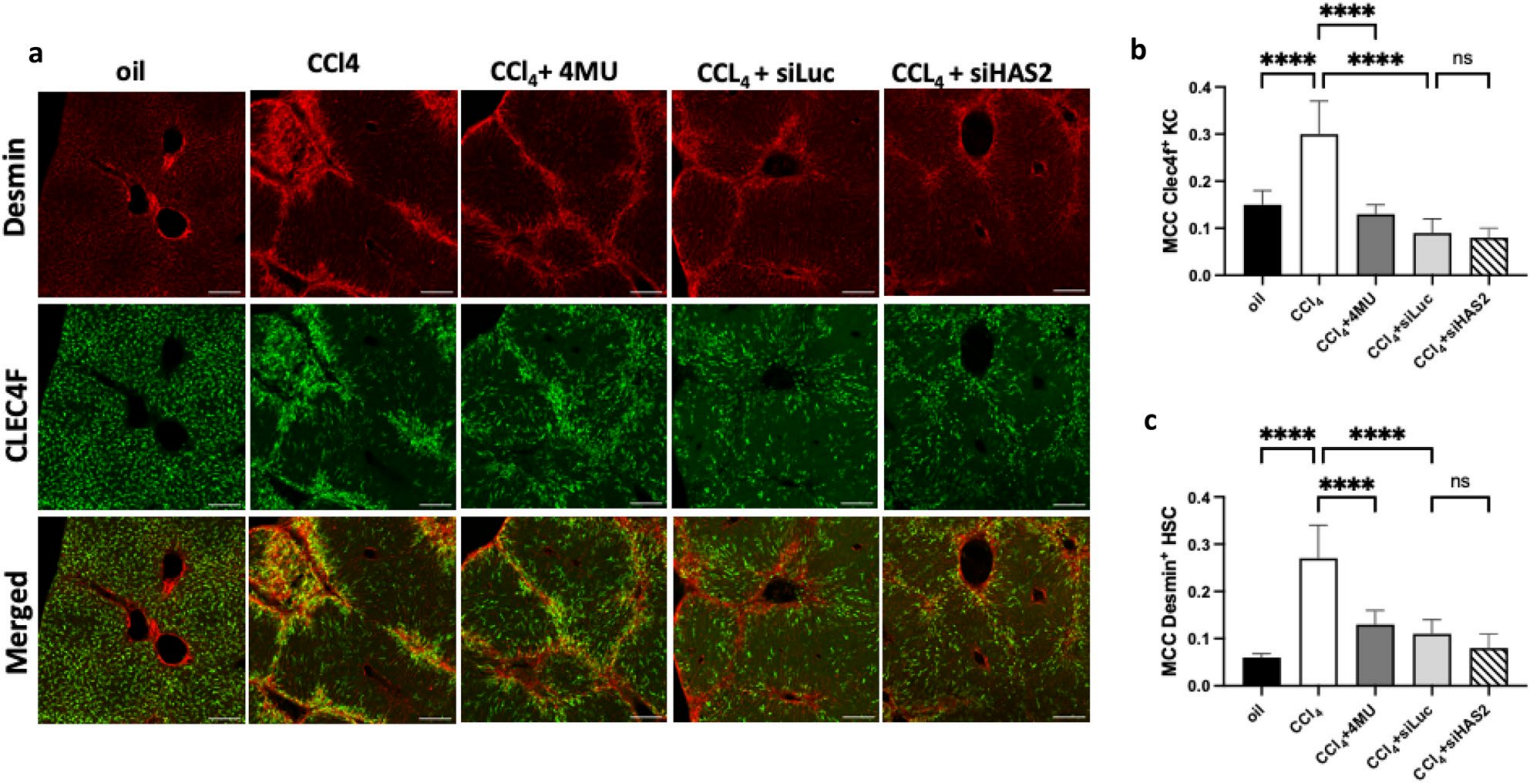
Immunofluorescent image analysis of colocalisation of HSCs and KCs in mice liver sections. (**a**) Representative images of Desmin-positive HSCs (red colour) and Clec4f.-positive KCs (green colour) in mice liver sections, scale bar- 200 µm. (**b**) and (**c**) Quantification of association using MCC. Data are presented as mean ± SD, n = 4 One-way ANOVA ***p < 0,001.

The development and progression of fibrosis is primarily influenced by Kupffer cells (KCs) and hepatic stellate cells (HSCs). Early signals for HSCs activation most likely originate from tissue-resident KCs in response to tissue damage. Both cell types migrate to the injury site; thus, we investigated the colocalisation of HSCs and KCs using immunofluorescence (Fig. 2). In contrast to qHSCs, activated HSCs have been shown to upregulate the expression of Desmin and Vimentin proteins^45–47^. Using a KC-specific marker, C-Type Lectin Domain Family 4 Member F (Clec4f) and HSC marker Desmin, we observed that HSCs and KCs are in direct contact (Fig. 2a). Quantifying colocalisation of Clec4f-positive KC and Desmin-positive HSCs using Manders colocalisation coefficient (MCC) showed that only 6% of Desmin-positive HSCs were found near 15% of Clec4f-positive KCs in the non-fibrotic control. However, after inducing fibrosis, the number of both cell types in close contact significantly increased (30% of HSCs and 27% of KCs) (Fig. 2b, c). Additionally, the estimation of cell type proportion in deconvoluted bulk RNAseq data showed an increased proportion of Kupffer cells in the fibrosis model (Fig. 9b). Treatment with 4MU, siHAS2-LNP, and siLuc-LNP significantly reduced the overlap between HSCs and KCs in the scar area, reflected by a reduction in both MCC coefficients.

### Effect of 4MU and HAS2 knockdown on HSC activation

**Figure 3:**
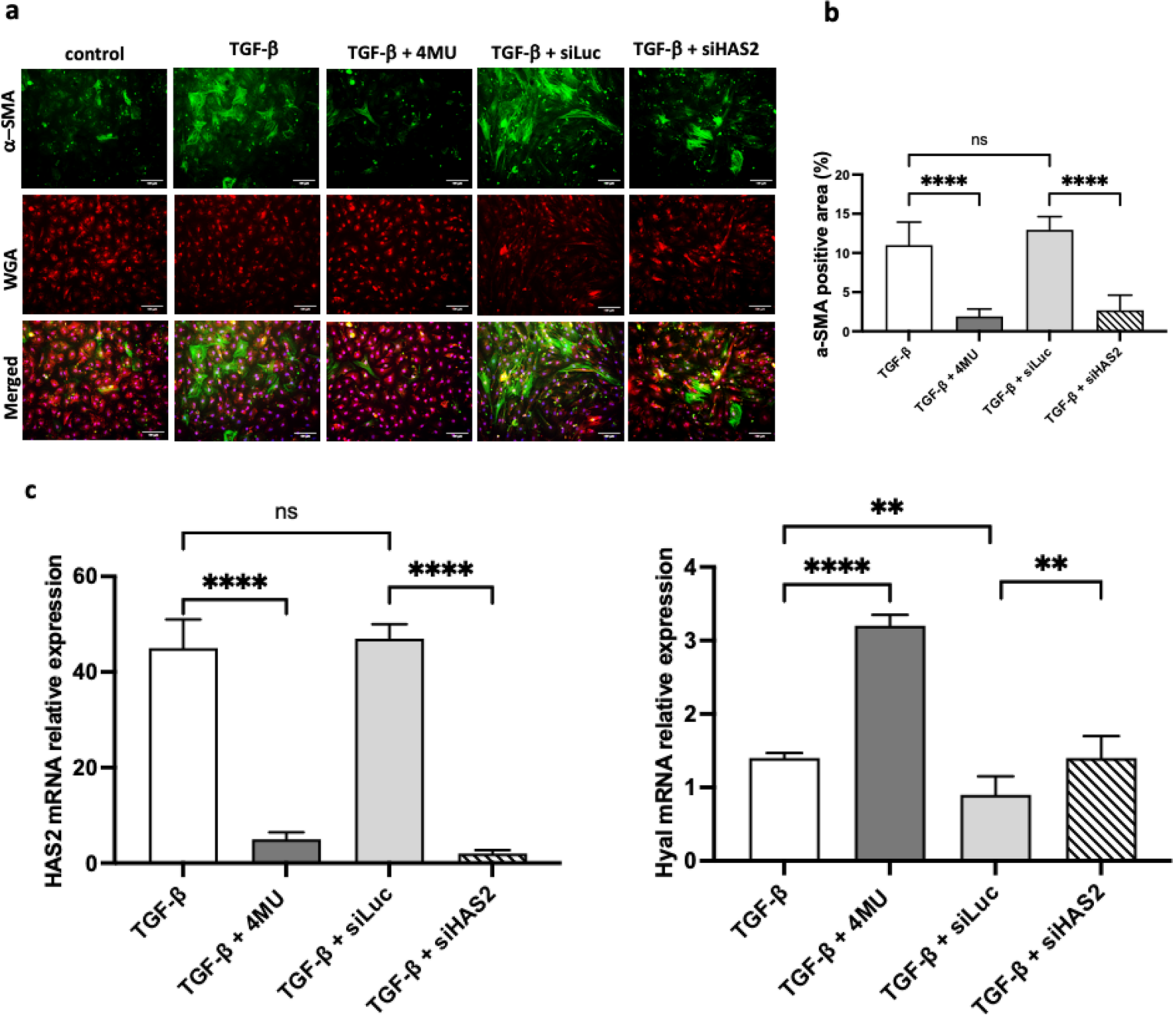
Immunofluorescent image analysis of activated HSC cells. (**a**) 4MU and siHAS2-LNP treatments inhibit TGF-b induced HSCs activation. Representative images of a-SMA positive (green colour) activated HSCs. Wheat germ agglutinin (WGA: red colour) shows the plasma membrane of cells, scale bar- 100 µm. (**b**) Quantification of a-SMA positive area in activated HSCs, n = 15 field of view per group (**c**). HAS2 and Hyal1 analysis of relative expression of mRNA in TGF-b activated HSCs. Data are presented as mean ± SD, n = 4 One-way ANOVA, ****p < 0.0001, ***—p < 0,001, **—p < 0,01, *—p < 0,05.

HAS2 is the primary isoform responsible for hyaluronic acid synthesis. It is predominantly expressed in activated hepatic stellate cells (α-SMA^+^ HSCs) in the liver^48^. In vivo, we observed a decrease in the HA deposition after administering our non-targeting siRNA control (siLuc-LNP). To elucidate whether the observed in vivo effect was due to a nonspecific response to the cationic lipid nanoparticles (LNP) used for siRNA delivery, we modelled HSCs activation in vitro using TGF-β. To accomplish this, the isolated HSCs were transfected with cationic lipid encapsulated siRNA for luciferase and HAS2 for 24 h, and then 20 ng/ml TGF-β and 4MU (0.25 mM) were added. After 72 h, cells were fixed and stained with antibodies to α-SMA, a marker of activated HSCs. Both siHAS2 and 4MU significantly reduced α-SMA expression associated with HSCs differentiation into myofibroblast. However, siLuc-LNP did not affect α-SMA expression in TGF-β activated HSCs (Fig. 3a, b). This demonstrates the target-specific effect of siHAS2 on the cellular level and underpins inflammation exacerbation (IE) as the primary phenomena responsible for the antifibrotic effect observed in siLuc-LNP treatment in vivo.

Next, the mRNA levels of enzymes responsible for HA production and degradation, HAS and hyaluronidase (Hyal), were quantified by qPCR. The results indicate that TGF-β-activated HSCs expressed significantly higher levels of HAS2 than non-activated HSCs (Fig. 3c). Treatment with siHAS2 and 4MU led to a significant decrease in the relative quantity of HAS2 mRNA. Interestingly, 4MU treatment induced the expression of Hyal1 in non-activated HSCs, but when activated with TGF-β, the expression levels of Hyal1 returned to control levels. In contrast, siHAS2-LNP transfection decreased Hyal1 expression in activated HSCs compared to non-transfected activated HSCs. Furthermore, the mRNA levels of HAS2 and Hyal1 in siLuc-transfected cells were comparable to non-transfected TGF-β-activated control. Taken together, these findings highlight the interplay between the expression of HA-producing and HA-degrading enzymes. The upregulation of HAS2 in TGF-β-activated HSCs contributes to the increased production of HA, while the downregulation of Hyal1 leads to its accumulation. The differential effects of 4MU and siHAS2-LNP treatments on the expression of these enzymes further emphasise the intricacies of their regulation and the need for further investigation.

**Figure 4:**
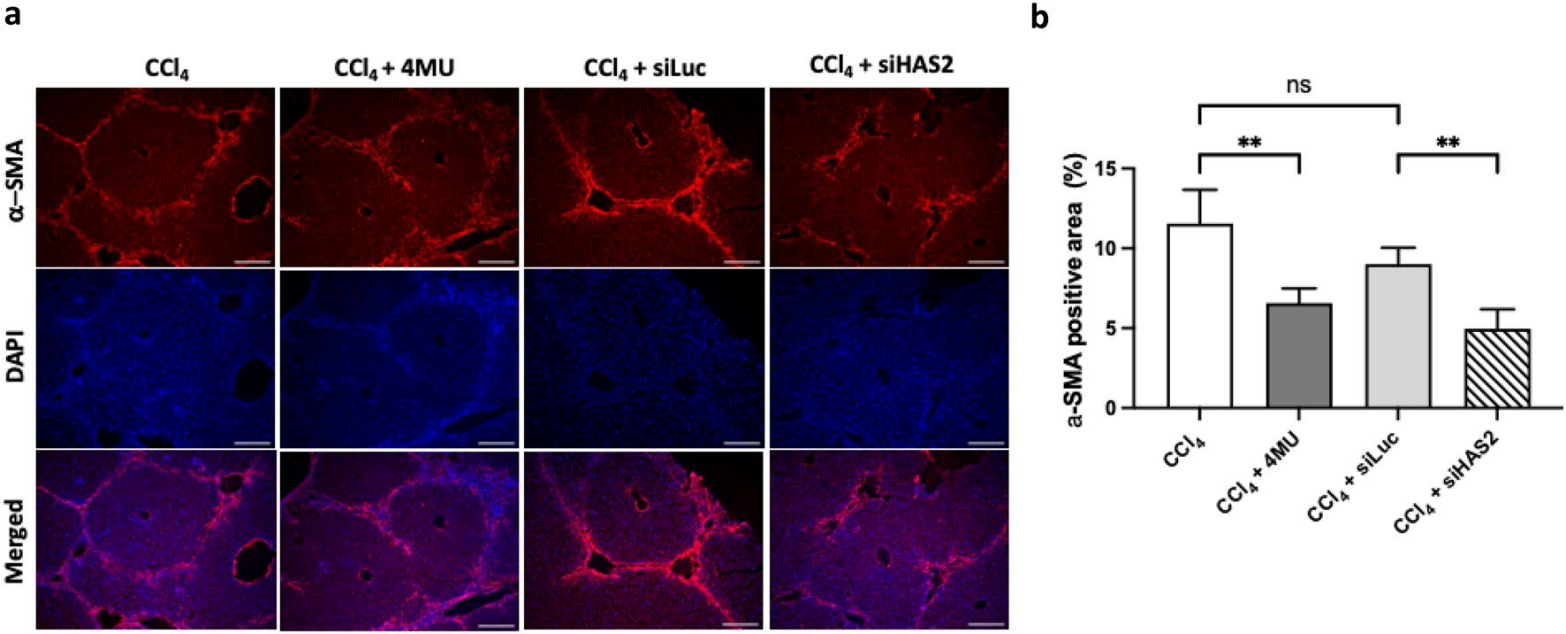
Immunofluorescent image analysis of α-SMA positive area in CCl_4_ treated mice liver sections. (**a**) Representative images of α-SMA positive (red colour) area in fibrotic mice treated with 4MU, siLuc and siHAS2. Blue colour (DAPI dye) shows the nucleus of parenchyma cells, scale bar 100um. (**b**) Quantification of α-SMA positive area in tissue. Data are presented as mean ± SD, n = 4 One-way ANOVA, **—p < 0,01, ns-non-significant.

Furthermore, we stained liver sections with anti-α-SMA antibody (Fig. 4a, b) and observed that siHAS2 significantly inhibits α-SMA expression in vivo, validating observations made from in vitro analysis.

### *siHAS2-LNP ameliorate CCl*_*4*_*-induced hepatic injury*

**Figure 5:**
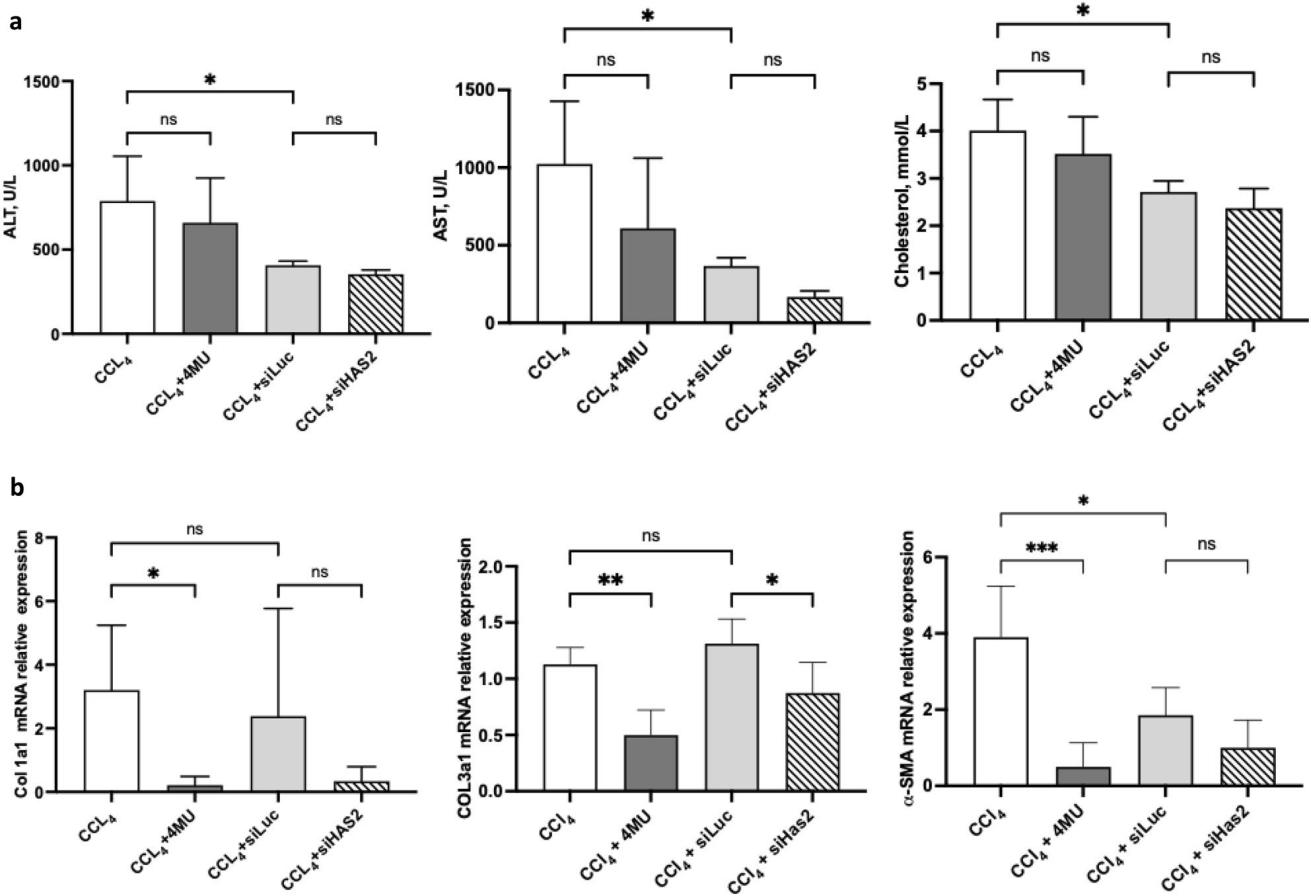
Fibrotic mice liver analysis after two weeks of CCl_4_ treatment. (**a**) Liver function tests: AST, ALT, and cholesterol in serum of mice and hepatic expression of canonical markers of fibrosis after CCl_4_ treatment. (**b**) Relative mRNA expression analysis of Col1a1, Col3a1, and α-SMA in treatment groups. Data are presented as mean ± SD, n = 4. One-way ANOVA; ***—p < 0,001, **—p < 0,01, * -p < 0,05.

Liver function tests and qPCR of canonical markers of liver fibrosis were investigated among the groups (Fig. 5). An ordinary One-way ANOVA showed marked upregulation of aspartate aminotransferase (AST) and alanine aminotransferase (ALT) upon CCl_4_-mediated hepatic damage. Quantifying AST and ALT levels in the blood serum of mice administered with 4MU demonstrated that 4MU did not prevent initial hepatocyte damage induced by toxic derivatives of CCl_4_. Both siHAS2-LNP and siLuc-LNP induced a comparable reduction in serum AST and ALT levels (Fig. 5a).

It has been observed that patients with fibrosis have decreased lipid metabolism and lower serum lipids^49^. Thus, we quantified cholesterol (Fig. 5a) and triglycerides (Supplementary material Fig. S2). Cholesterol levels in the blood sera of animals treated with 4MU and siHAS2-LNP remained low, while triglyceride levels were elevated by 4MU treatment. Additionally, our transcriptome analysis revealed that the upregulated differentially expressed genes (DEGs) after 4MU administration were involved in triglyceride anabolism and cholesterol metabolism (Fig. 10b). qPCR showed a significant upregulation of α-SMA, Col1a1, and Col3a1, typical markers of HSC activation in fibrosis (Fig. 5b). However, treatment with 4MU significantly lowered Col1a1, Col3a1, and α-SMA mRNA levels.

Similarly, treatment with siHAS2-LNP downregulated the expression of both Col1a1 and α-SMA but did not alter the transcript level of Col3a1. Although siLuc-LNP did not significantly alter the expression of Col1a1 and Col3a1, we observed a marked downregulation of the α-SMA mRNA level. This effect was completely absent in the cell culture of activated HSC (Fig. 3b).

### 4MU and siHAS2-LNP treatment inhibits HA deposition

**Figure 6:**
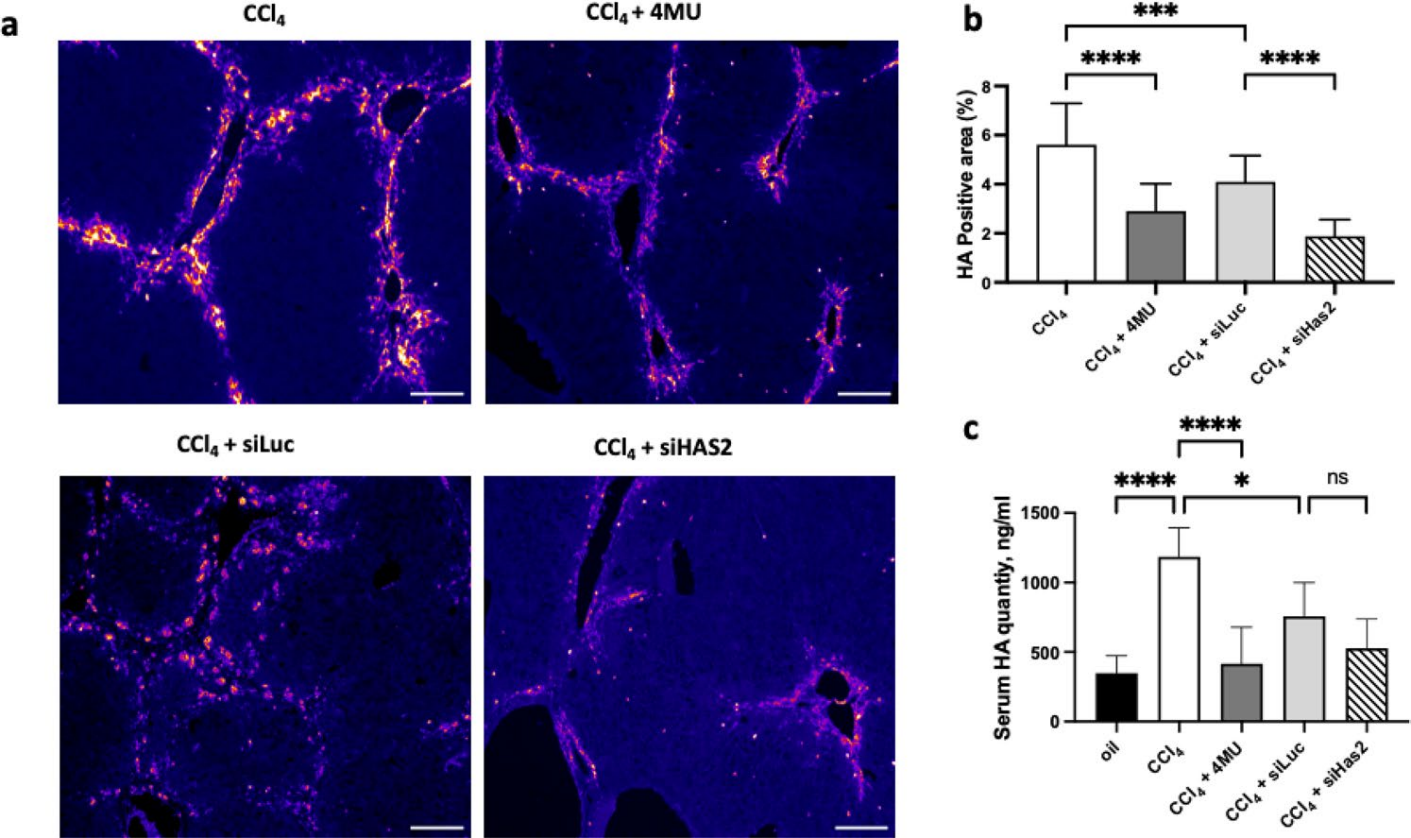
Immunofluorescent image analysis of HA deposition in liver fibrosis. (**a**) Representative images of hyaluronic acid binding protein (HABP)-stained (pink-red colour) liver sections, scale bar—100 μm. (**b**) Quantification of HA-positive area. (**c**) HA concentration in the serum of mice with CCl_4_-induced fibrosis treated with 4MU, siLuc-LNP and siHAS2-LNP. Data are presented as mean ± SD, n = 5. One-way ANOVA, ***—p < 0.001, ****—p < 0.0001.

To determine the enzymatic activity of HAS2, we quantified the amount of HA produced by HAS2 (Fig. 6a, b). HSCs have been demonstrated to be the primary source of the myofibroblast pool during hepatic fibrosis. These myofibroblasts are responsible for the production and deposition of HA and other components of the ECM^48^. Figure 6 highlights that the quantity of HA is elevated after chronic exposure to CCl_4_ but is lowered upon treatment with both 4MU and siHAS2-LNP, with siHAS2-LNP exerting the most potent down-regulation. In the absence of hepatic damage, HA quantity is low. The serum HA level was elevated nearly four-fold in CCl_4_-treated mice (Fig. 6c) but was restored to control levels upon administration of 4MU.

Treatment with siHAS2-LNP or 4MU reduced HA deposition in zone 1 of liver lobules. siLuc-LNP had a similar effect but to a lesser degree than specific HAS2 knockdown with siHAS2-LNP.

### Hepatic transcriptomic signature associated with CCl_4_ induced fibrosis

**Figure 7:**
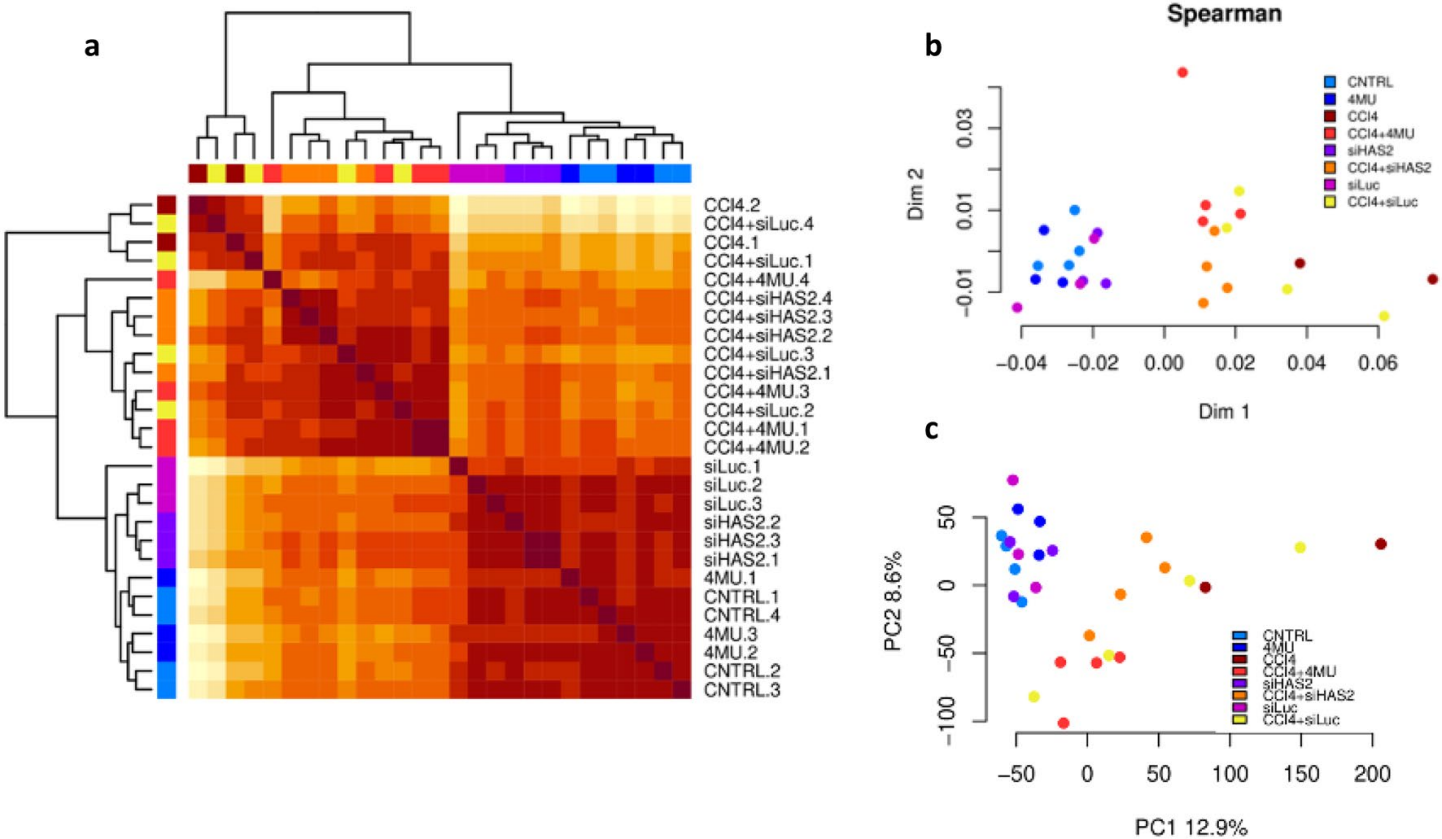
Exploratory analysis of transcriptome from the total liver of mice from eight different treatment groups, control, 4MU, siLuc-LNP, siHAS2-LNP, CCl_4,_ CCl_4_ + 4MU, CCl_4_ + siLuc-LNP and CCl_4_ + siHAS2-LNP. (**a**) Hierarchical clustering of biological replicates based on Spearman correlation of transcriptomic signatures of non-treated and CCl_4_ treated mice liver samples. The brown-to-white colour gradient indicates a high-to-low level of correlation. (**b**) Multidimensional scaling (MDS) based on Spearman correlation. (**c**) Principal Component Analysis (PCA) plot of all samples. Each point represents a sample, and colours indicate different experimental groups, as shown in the legend provided.

To ensure accuracy, we first tested all replicates from all eight experimental groups for self-consistency. We then performed hierarchical clustering (Fig. 7a) and multidimensional scaling (MDS) (Fig. 7b) using Spearman correlation between samples. Additionally, we conducted a principal component analysis (PCA), where PC1 and PC2 accounted for 12.9% and 8.6% of the variance, respectively (Fig. 7c). Our verification through these different methods revealed that the CCl_*4*_-treated mice were distinct from the non-CCl_*4*_-treated mice. The most extensive diversity was observed in fibrotic mice administered with siLuc-LNP.

In order to understand the molecular mechanism responsible for hepatic damage and fibrosis caused by CCl_4_, we conducted bulk RNA-seq analysis to compare the gene expression patterns of CCl_4_-treated and non-treated mice. The results showed that there were 6689 DEGs, with 3481 upregulated and 3201 downregulated. The treatment with CCl_4_ led to a significant increase (log2 FC > 2, p adj value < 0.05) in approximately 418 genes, with the most notable increase taking place in genes that regulate DNA replication and cell cycle control (Smc2, Gins1, Map4k3, Zfp532, Pign, Gtse, Kifc1, Pard6b, Myo9b), cell motility and adhesion (Lratd1, Ppfia1, Gldn) and inflammation (Akap10, Tabl) (see Supplementary Table S1). We observed the upregulation of Fkbp10, a molecular chaperone found in the ER that fuels the progression of hepatic fibrosis. Fkbp10 plays a crucial role in the post-translational modification of collagen. It ensures normal collagen secretion, stable inter-molecular crosslinking, and its deposition in the ECM^50–52^*.*

In our previous study, we observed a dramatic increase in FSTL1, a widely expressed glycoprotein secreted in response to CCl_4_ treatment. FSTL1 has been implicated in the progression of renal and hepatic fibrosis, and its transient knockdown in vivo resulted in a muted expression of Col1a1 mRNA and inhibited collagen deposition^42,53^. Consistent with our previous findings, FSTL1 mRNA expression was upregulated, although not as dramatically. Interestingly, Egr2, a cytokine downstream of TGF-β signal transduction^54^, was also highly induced in response to CCl_4_ treatment. Overexpression of Egr2 in skin fibroblasts showed a dose-dependent induction of expression of type I collagen and α-SMA protein levels^55^. It is speculated that Egr2 fuels the progression of fibrosis by modifying macrophage localisation and HSC expansion^53^. We also noted an upregulation in the expression of JAZF1, known for its role as a transcriptional corepressor of the orphan nuclear receptor NR2C2. JAZF1 is implicated in the transcriptional activation of nicotinamide phosphoribosyltransferase (NAMPT) by promoting PPARα and PPARδ expression. Additionally, it regulates lipid metabolism and glucose homeostasis^56^.

CCl_4_ significantly down-regulated several genes associated with cellular response to members of the mouse urinary protein (MUP) family (Mup2, Mup9, Mup10, Mup11, Mup13, Mup17). The expression of MUP proteins is known to be induced by androgens. MUPs are physiologically relevant, as they bind and carry small hydrophobic molecules, such as steroid hormones, lipids, and pheromones. Furthermore, MUP expression is affected by insulin resistance and has been associated with energy expenditure. MUPs were also recently reported to be some of the most down-regulated genes in response to CCl_4_ treatment of male C57BL/6N mice^57,58^. Several oxidoreductases from the CYP450 superfamily (Cyp3a41a, Cyp3a41b, Cyp3a16, Cyp2c69, Cyp2a5, Cyp2c38) involved in xenobiotic and lipid metabolism were prominently downregulated. Additionally, two genes from the sulfotransferase family (Sult2a3 and Sult3a1) were markedly downregulated. Sulfotransferases play a role in the sulfonation of steroids and bile acids, converting them into easily excretable hydrophilic water-soluble sulfate conjugates^59^. Other transferases were also downregulated, including those involved in amino acid metabolism and transport (Glutamine synthetase: glul, Ornithine Aminotransferase 2: Oat, glutamate transporter 1: Slc1a2), fatty acid biosynthesis (ELOVL Fatty Acid Elongase 3: Elovl3), and histone acetylation (N-acetyltransferase family 8 member 7: Nat8f7).

**Figure 8:**
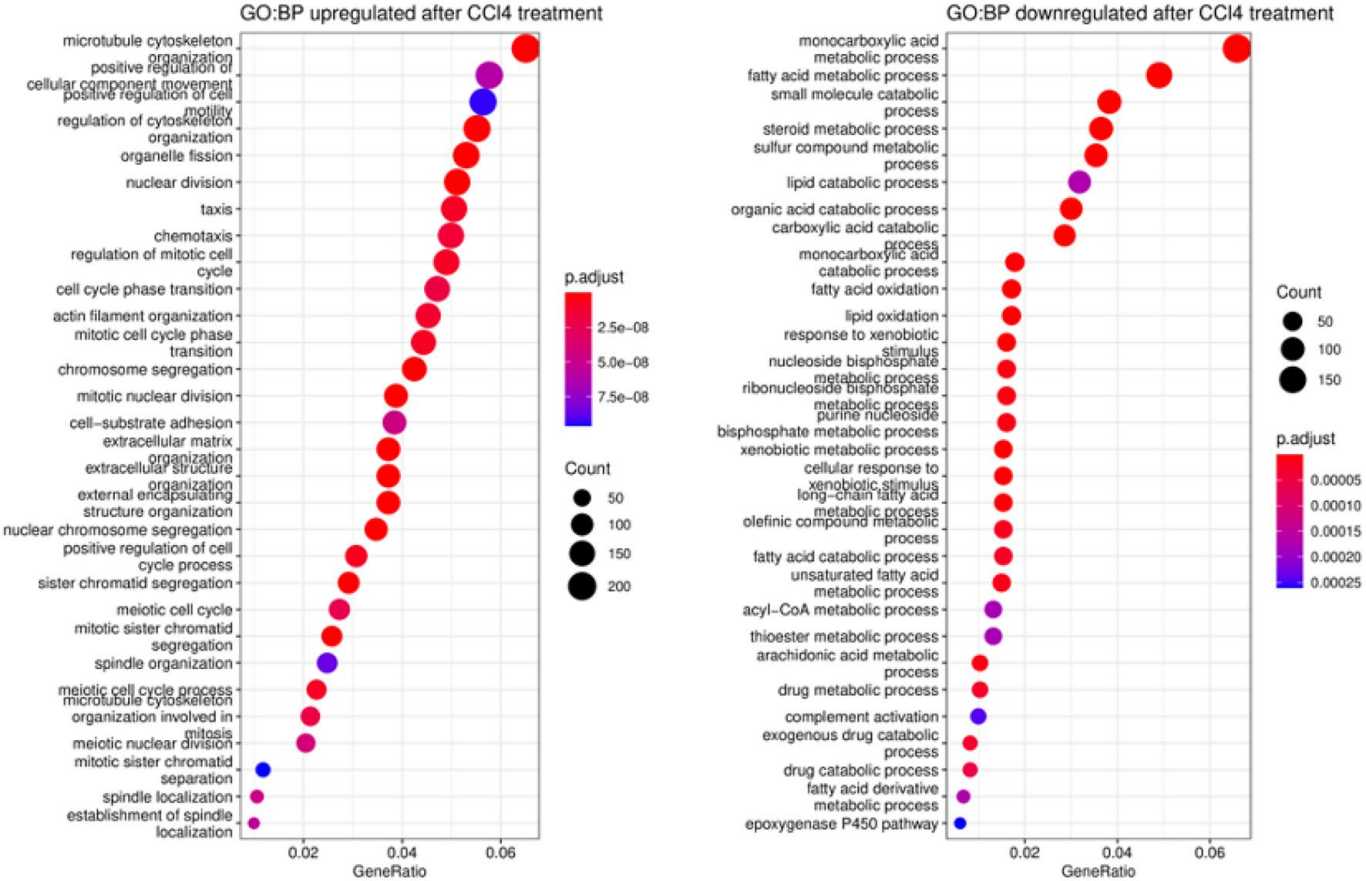
Dot plot enrichment maps of CCl_4_ upregulated and downregulated DEGs. Gene Ontology biological pathway (GO BP) associated with upregulated and downregulated DEGs. The colour of the dot is indicative of p.adj, and size represents the number of DEGs in the related pathway.

The DEGs were subjected to Gene Ontology biological pathways (GO BP) enrichment analysis to determine their biological functions (Fig. 8). The results showed that the downregulated DEGs were primarily associated with the homeostatic functions of hepatocytes, including lipid, steroid, xenobiotic and drug metabolism, and solute-coupled transport. On the other hand, the upregulated DEGs were enriched in pathways related to DNA replication and repair, mitosis, cell division, ECM organisation, and cell motility, which are typically associated with myofibroblasts.

### Inferred cellular heterogeneity at fibrosis milieu from bulk RNA-seq data

**Figure 9:**
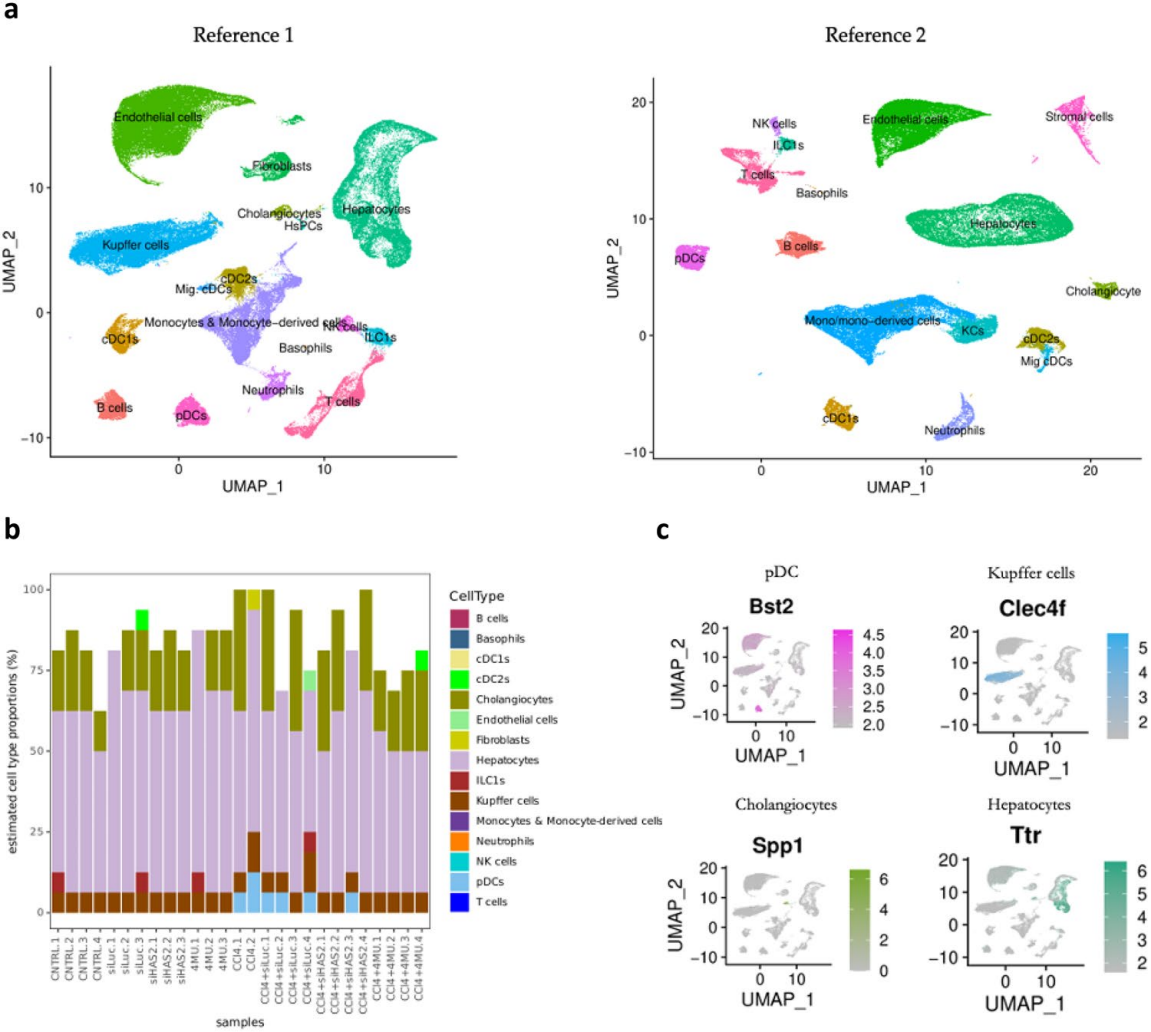
Cell composition deconvolution from total liver RNA-Seq data from all experimental groups. (**a**) Combined and integrated single-cell reference data sets extracted from liver cell Atlas database. (**b**) Predicted change of cell type proportions across different treatments. (**c)** Validation of reference cell annotation by cell type-specific marker genes for Plasmacytoid dendritic cells (pDCs), Kupffer cells, Cholangiocytes, and Hepatocytes. Normalised expression values are shown.

To determine the proportions of different cell types present in our bulk RNA-seq data, we utilised two scRNA-seq datasets to create a reference expression profile. However, due to a lack of scRNA-seq data on CCl_4_-mediated fibrosis, we instead used two different datasets as references: one from standard diet-fed mice (Fig. 9a, Reference 1) and the other from mice with NAFLD (Fig. 9a, Reference 2). We compared the two reference datasets by plotting their cell-type similarity matrix and computing their condition number (k). Our analysis revealed that Reference 1 had a lower condition number than Reference 2, indicating more stable and reliable results. Consequently, we selected Reference 1 to estimate the cell type proportions in our bulk RNA-seq data. We conducted a deconvolution assay using the granulator R package following the methods described in^60^ (Fig. 9b). To validate the cell annotation of the reference dataset, we performed a correlation analysis of the deconvoluted proportions using the canonical expression markers of significant liver cell types. We observed consistent and cell type-specific expression patterns for Bone Marrow Stromal Cell Antigen 2 (Bst2), C-Type Lectin Domain Family 4 Member F (Clec4f), Secreted Phosphoprotein 1 (Spp1) and Transthyretin (Ttr) in cell types annotated as pDCs, Kupffer cells, cholangiocytes, and hepatocytes, respectively (Fig. 9c).

The key observation from this computational approach is the appearance of plasmacytoid dendritic cells (pDCs) in CCl_4_ and CCl_4_-siLuc-LNP-treated mice, which are absent in non-fibrotic mice. Previous studies have shown that small fragments of HA drive the activation and maturation of dendritic cells (DCs) via the TLR4 signal transduction pathway involving NFκβ^61^. pDCs are absent in fibrotic mice treated with 4MU and siHAS2-LNP, suggesting their role in inflammation. The appearance of pDC in fibrotic mice is accompanied by an increased proportion of Kupffer cells, the hepatic resident macrophages, which also drive inflammation. Furthermore, an increased proportion of cholangiocytes was observed in all CCl_4_-administered mice compared to non-CCl_4_-administered mice, possibly explained by dysregulation of bile metabolism and the resultant accumulation of bile acid correlates with hepatic damage. The proportion of hepatocytes in CCl_4_-4MU mice is significantly lower than in the other groups. This observation is characteristic of the cytostatic nature of 4MU, which has been demonstrated both in vitro and in vivo^42,62,63^. 4MU inhibits the proliferation of hepatocytes and other cell types in the fibrotic milieu, particularly the fibroblast. Although this inhibition could be viewed as a double-edged sword, the overall effect is beneficial as it orchestrates the fine-tuned balance between fibrosis regression and the progression of liver regeneration. Fibroblasts, sinusoidal endothelial cells, and type 2 conventional dendritic cells (cDC2) make up approximately 5% of the liver cell population, and their predicted proportions varied from group to group, likely due to the uncertainty of the prediction model. Overall, these observations indicate a distinct signature and dynamic cell–cell crosstalk during fibrosis.

### *Transcriptomic analysis underpins 4MU alleviation of CCl*_*4 -*_*induced fibrosis in mice*

The hepatotoxicity of CCl_4_ results from its metabolic activation to reactive free radicals by CYP2E1. These radicals covalently bind cellular macromolecules and induce enhanced peroxidation of unsaturated fatty acids of phospholipids, causing damage to cellular membranes and ultimately leading to apoptosis or necrosis. The DAMPs from apoptotic and or necrotic hepatocytes are detected by resident macrophages, which promote inflammation^64–67^. As a result of the inflammation, qHSCs get activated and transdifferentiate to myofibroblasts. These myofibroblasts produce ECM, predominantly collagen type I and III, accumulating to form the fibrotic scar. 4MU is a known inhibitor of HA synthesis. We observed a marked upregulation of Col1a1, Col3a1, and α-SMA mRNA levels in the CCl_4_ group (Fig. 5b), all of which were significantly reduced by 4MU treatment_._ Treatment with siHAS2-LNP also significantly downregulated the expression of Col1a1 and α-SMA but had no significant effect on Col3a1.

**Figure 10:**
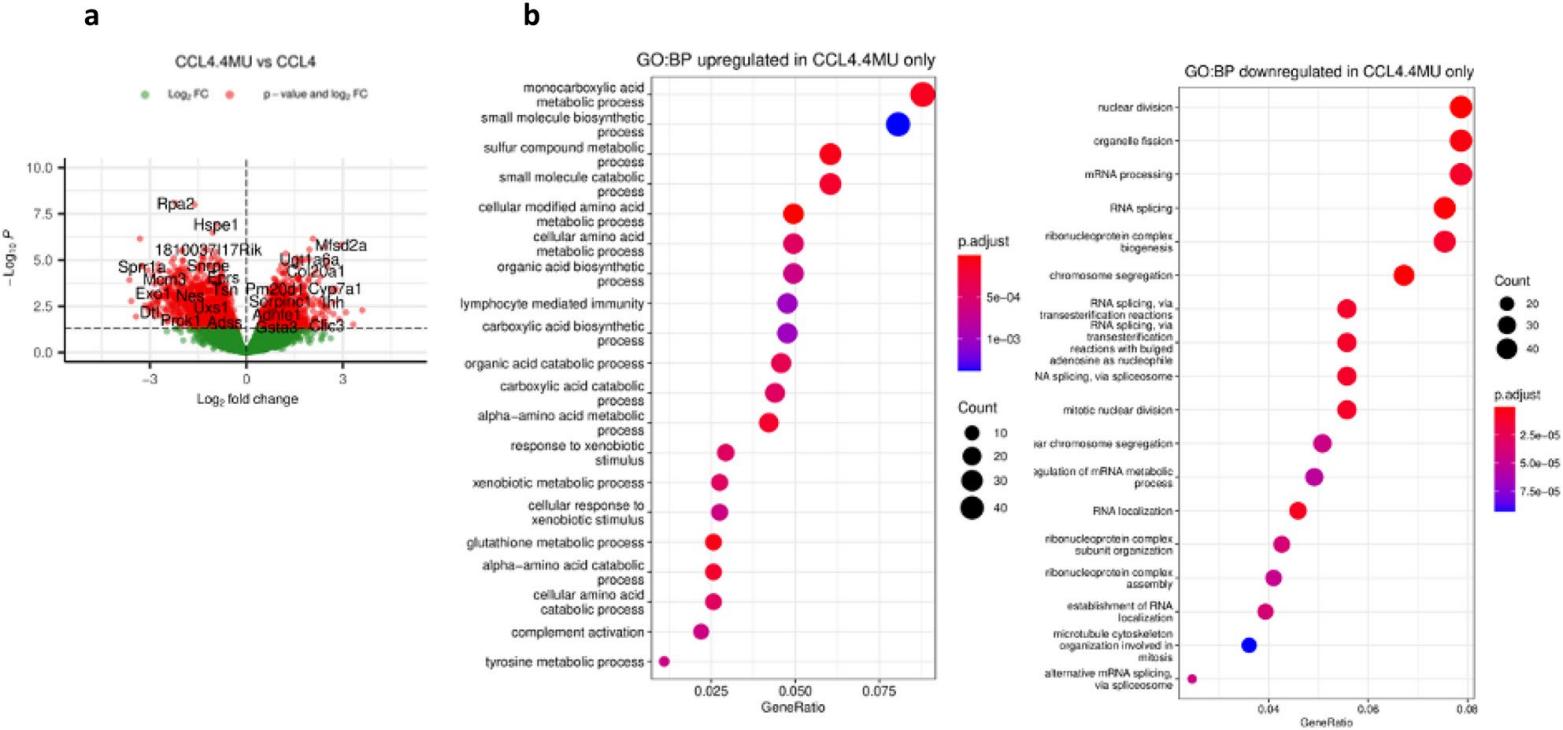
Volcano plot and Dot plot enrichment maps of DEGs from CCl_4_ vs CCl_4_ + 4MU samples. (**a**) The Volcano plot compared the DEGs between CCl_4_ and CCl_4_ + 4MU treated mice. The p-values < 0.05, and logFC ≥ 1 and logFC ≤ –`1 are in red dots; the significant DEGs satisfying both values are in red dots, and some are indicated with gene names. The genes that are upregulated in the array are on the right panel, and the downregulated ones are on the left panel of the plot. (**b**) Dot plot enrichment map showing GO BP associated with upregulated and down-regulated DEGs, respectively. The colour of the dot is indicative of p.adj, and size represents the number of DEGs in the related pathway.

To investigate the protective effect of 4MU against CCl_4_-induced liver fibrosis in mice, we compared the transcriptomic signatures of mice with untreated fibrosis (CCl_4_ group) to those which received 4MU treatment (CCl_4_-4MU group). Transcriptomic analysis revealed 988 upregulated and 1264 downregulated DEGs. We conducted GO BP enrichment analysis on the upregulated gene set and filtered the results by fold enrichment. The top 10 biological processes were related to amino acid catabolism (tyrosine, L-phenylalanine), chylomicron remnant clearance, purine deoxyribonucleoside metabolism, cholesterol catabolism, phospholipid efflux, triglyceride anabolism, glutathione metabolism, epoxygenase P450 pathway, complement activation, bile acid metabolism, autophagy of mitochondrion, and gluconeogenesis (Fig. 10).

The downregulated genes were mapped using the Search Tool for the Retrieval of Interacting Genes/Proteins (STRING). The resulting protein–protein interaction network (PPI) was then exported to Cytoscape to identify significant modules using the Molecular Complex Detection (MCODE) plug-in. The selection criteria were: MCODE scores ≥ 4; degree cut‑off = 2; node score cut‑off = 0.2; maxdepth = 100; and k‑score = 2. The analysis produced three modules that were identified as the most significant. Functional annotation and enrichment analysis of the most significant module, which had 36 nodes, 541 edges, and an MCODE score of 36, revealed that the biological process primarily affected was the cell cycle, particularly the processes of G2/M transition, chromosome segregation, chromatid separation, mitotic cytokinesis spindle assembly and localisation. These findings provide further evidence of the cytostatic effect of 4MU, which has been well-documented in several studies^42,62,63^.

The second most significant module was associated with RNA processing machinery, including messenger ribonucleoprotein complex assembly, alternative splicing via spliceosome, localisation, 3’ UTR-mediated mRNA stabilisation and ribosome biogenesis. The genes in module 3 were enriched in translation, formation of cytoplasmic translation initiation complex and regulation of protein metabolism. 4MU inhibited the formation of transcriptionally inert chromatin by downregulating genes that promote the formation of repressive chromatin structures (Smchd1, Ezh2, Hat1, Rif1, Hells). Moreover, 4MU downregulated the expression of genes associated with transcription-inhibitory histone ubiquitination (RNF168, Bard1, Rad51, Pcgf5, Brca2, Brca1). Although 4MU induced the formation of chromatin structures that favour transcription, it also induced global repression of gene expression by inhibiting mRNA export from the nucleus, splicing via spliceosome, spliceosomal complex assembly, and maturation of 5.8S rRNA. At the protein level, 4MU downregulated translation and post-transcriptional regulation of gene expression, as well as SRP-dependent cotranslational protein targeting to the membrane.

Our results collectively demonstrate that 4MU protects the liver by upregulating lipid, xenobiotic, glutathione, and small molecule metabolic processes, thereby restoring the liver’s normal function. Moreover, it prevents the depletion of cellular resources by inhibiting cell cycle and protein biogenesis by disrupting RNA processing and translation machinery. Histological analysis, RNAseq, liver function test and qPCR of key fibrosis markers confirm that 4MU exhibits antiproliferative and antifibrotic activity, providing protection to the liver against CCl_4_-mediated damage.

### siRNA-mediated knockdown of HAS2 inhibits fibrosis by regulating ECM

**Figure 11:**
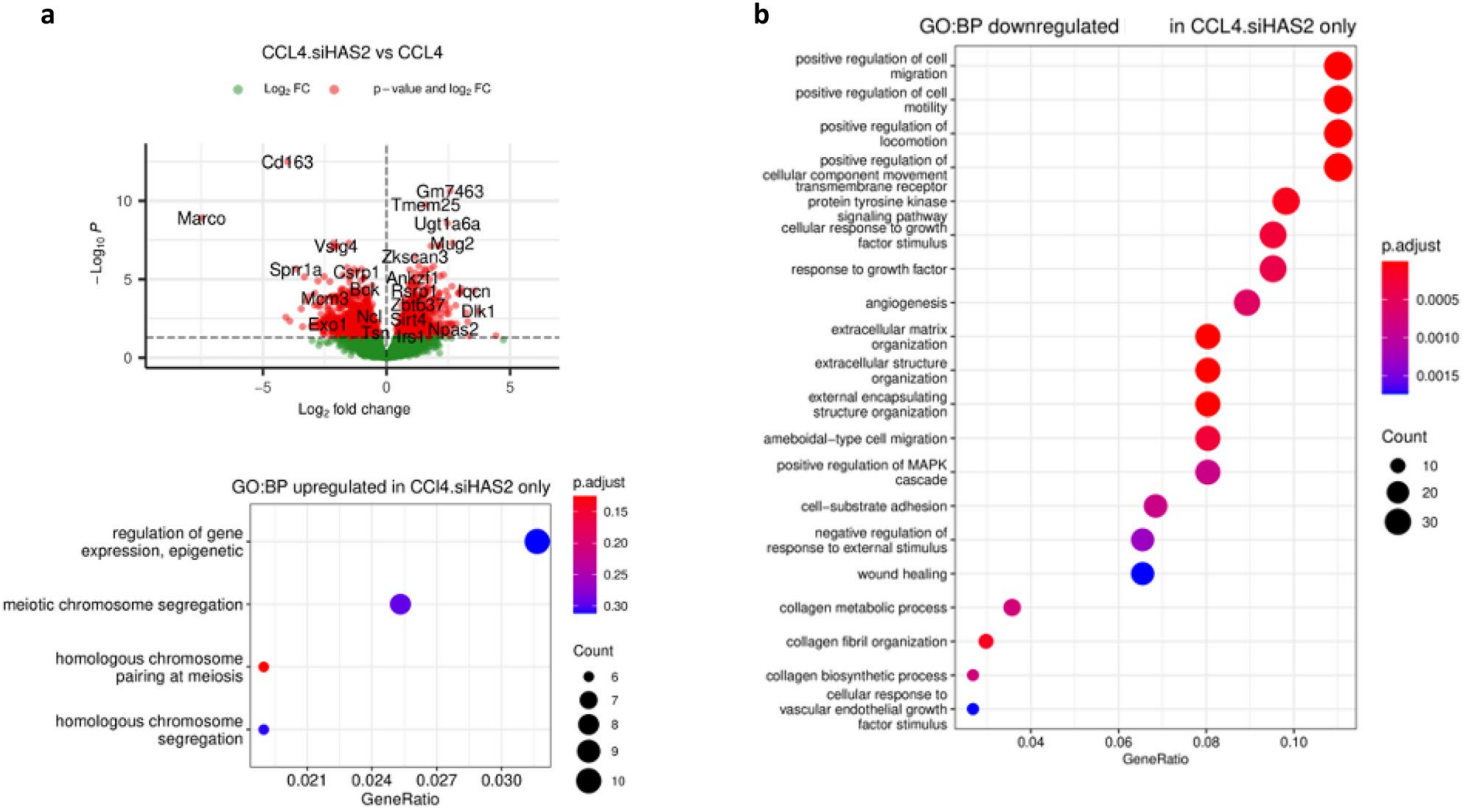
Volcano plot and Dot plot enrichment maps of DEGs from CCl4 vs CCl4 + siHAS2-LNP. (a) The Volcano plot compared the DEGs between CCl4 and CCl4 + siHAS2-LNP treated mice. The p-values < 0.05, and logFC ≥ 1 and logFC ≤ –`1 are in are in red dots; the significant DEGs satisfying both values are in red dots, and some are indicated with gene names. The genes that are upregulated in the array are on the right panel, and the downregulated ones are on the left panel of the plot. (b) Dot plot enrichment map showing GO BP associated with upregulated and down-regulated DEGs, respectively. The colour of the dot is indicative of p.adj, and size represents the number of DEGs in the related pathway.

HA is an essential component for fibroblast function and wound healing. In vivo, at sites of inflammation, high-molecular-weight (HMW) HA (size 1 × 10^6^ kDa) can be degraded into low-molecular-weight (LMW) (size 2 × 10^5^ kDa) fragments through reactions with oxygen radicals and enzymatic hydrolysis. Similarly, pro-inflammatory cytokines, such as TNF-α, can stimulate the fragmentation of HMW HA produced by fibroblasts^68^. Our histological analysis of mouse liver tissue revealed that siHAS2-mediated knockdown reduced collagen fibre deposition. This finding aligns with our transcriptomic analysis, in which the 977 downregulated genes were involved in collagen biosynthesis, extracellular matrix assembly, collagen fibril organisation, wound healing, cell adhesion and migration pathways. The corresponding signalling pathways were also negatively affected by siHAS2-LNP, including the canonical transforming growth factor and semaphorin-plexin signalling pathways (Fig. 11).

Hypoxia plays an essential role in liver fibrosis by driving inflammation via NF-κβ signalling and stimulating transdifferentiation of qHSCs into myofibroblasts. The GO BP enrichment analysis revealed that some of the downregulated genes were associated with the cellular response to hypoxia, angiogenesis and mesenchymal cell differentiation.

Also, we observed enrichment in the somatic hypermutation of immunoglobulin genes (Mlh1, Msh6, Exo1 Msh2, Polq) and B cell activation (Lgals1, Msh2, Hspd1, Msh6, Atad5, Exosc3, Rnf168, Mlh1). It has been shown that HA stimulates B cell activation in vitro via its receptor CD44^69^. Intrahepatic B cells were found to be particularly sensitive to CCl_4_ as their numbers dropped approximately tenfold after one day following the treatment with CCl_4_. Furthermore, B cell–deficient mice had 6- to eightfold less interstitial collagen deposition than wild-type mice in CCl_4_ -induced hepatotoxicity, indicating that B cells are nonredundant participants in liver fibrosis progression^70^. Collectively, our findings demonstrate that knockdown of HAS2 and the subsequent depletion of HA ameliorates fibrosis by inhibiting collagen biosynthesis, cellular hypoxic response, and B cell activation.

### Common upregulated and downregulated biological pathways in the fibrosis background

**Figure 12:**
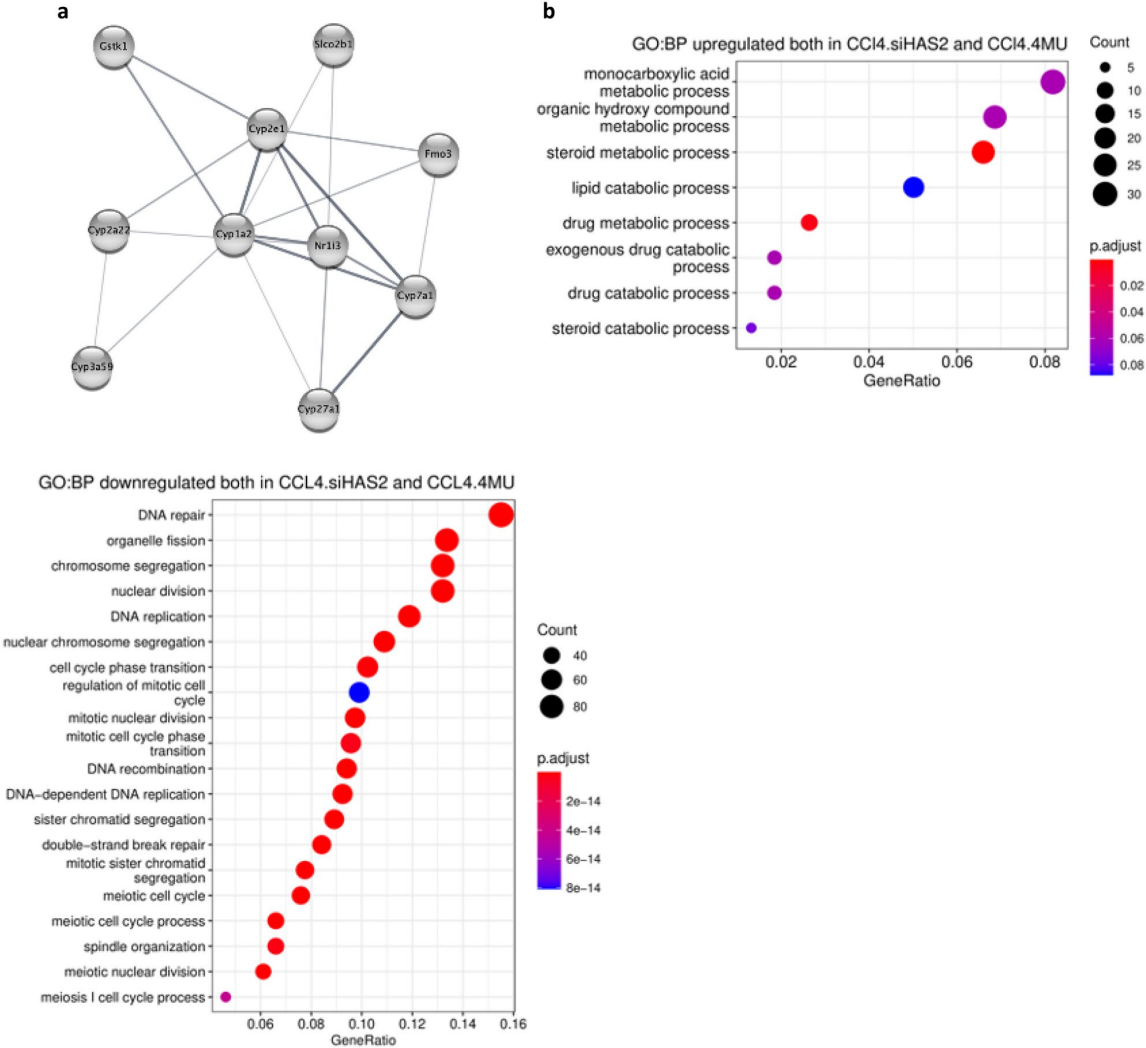
Network module built by MCODE and enrichment analysis of the common upregulated genes between CCl_4_ + siHAS2-LNP and CCl_4_ + 4MU. (**a**) The protein–protein interaction network of the most significant module, cluster 1, with 10 nodes, 20 edges and an MCODE score = 4.4. (**b**) Dot plot enrichment map showing GO BP associated with shared upregulated and downregulated genes. The colour of the dot is indicative of p.adj, and size represents the number of DEGs in the related pathway.

Transcriptomic patterns induced by 4MU treatment and HAS2 knockdown by siHAS2-LNP revealed a substantial number of common DEGs (Fig. 12). Both 4MU and siHAS2 ameliorate fibrosis by inhibiting HA production. We thus hypothesised that the shared DEGs were most likely to be the key genes essential for the regression of fibrosis. A total of 405 genes were upregulated, whereas 628 genes were downregulated.

The shared genes were mapped using STRING to construct a PPI network, which was exported to Cytoscape to identify the most significant modules using MCODE.

Based on the MCODE inclusion criterion, we identified three clusters, referred to as Cluster 1, Cluster 2, and Cluster 3, with an MCODE score ≥ 4 in the upregulated genes PPI network. Cluster 1, with MCODE score = 4.4, had 10 nodes and 20 edges, is illustrated in Fig. 12a. KEGG enrichment showed that cluster 1 genes are involved in drug metabolism, xenobiotic metabolism via CYP450, primary bile acid biosynthesis, and cholesterol metabolism.

Cluster 2 and 3 (Table 1) had an MCODE score of 4, with 4 nodes and 6 edges. The genes in Cluster 2 were primarily localized in the extracellular space and associated with innate immune response, complement activation via the classical pathway, and negative regulation of blood coagulation. Functional analysis of Cluster 3 using GO showed enrichment in macroautophagy, autophagosome assembly and mitophagy. Mitophagy is a mitochondrial quality control mechanism that allows for the selective removal of damaged mitochondria of the cell via the autophagy pathway^71^.

**Table 1:** MCODE Cluster 3 and 4 genes and functional annotation using GO BP enrichment analysis.

Cluster	Genes	Functional annotation
1	Cyp2e1, Cyp3a59, Gstk1, Cyp1a2, Cyp7a1, Cyp2a22, Fmo3, Nr1i3, Cyp27a1, Slco2b1	GO:0006706: Steroid catabolism GO:0016098: Monoterpenoid metabolism
2	Serping1, Masp2, C1ra, Kng1	GO:0006958: Complement activation, classical pathway GO:0030195: Negative regulation of blood coagulation
3	Park2, Rab7, Gabarapl1 Ambra1	GO:0016236: Macroautophagy

628 genes were downregulated in both CCl_4_-4MU and CCl_4_-siHAS2-LNP compared to the CCl_4_-only group. The MCODE plug-in was used to identify the most significant modules, and based on our inclusion criteria, 12 clusters with an MCODE score of ≥ 4 were identified. Cluster 1 PPI had an MCODE score of 96, with 105 nodes and 5013 edges, while cluster 2 had an MCODE score of 14, with 16 nodes and 104 edges. The GO BP enrichment analysis and functional annotation of Cluster 1 revealed that the top 10 GO BP, as ranked by false discovery rate (FDR), are the genes involved in the mitotic cell cycle, DNA conformation change, microtubule cytoskeleton organisation, cell cycle checkpoint, DNA recombination, Chromosome Condensation, DNA replication, meiosis 1, cell cycle phase transition, and histone phosphorylation. Regarding molecular function, most proteins exhibited binding activity towards enzymes, chromatin, microtubules, ATP, and DNA. Among them were proteins with ATPase, ssDNA helicase, kinase, and DNA translocase activities.

Cluster 2 functional enrichment analysis revealed that the genes were related to cells’ response to endoplasmic reticulum stress (ESR), Interleukin 7 pathway, and redox homeostasis (refer to Supplementary Table S4). Unresolved ER stress is known to induce apoptosis^72,73^. Our analysis indicates that both 4MU and siHAS2-LNP limited fibroblast activation by restricting the UPR and ERAD pathway usually elicited upon ER stress. Hepatocytes are the primary source of IL-7. The inhibition of IL-7 signalling was shown to mitigate inflammation in the liver. Furthermore, it is critical for the survival of naïve and memory T cell subsets^74^. It is essential for the reinforcement of cytotoxic T lymphocyte (CTL) activity during IFN-γ stimulation in vitro^75^.

Clusters 3,4,6,8 genes, much akin to cluster 1, were associated with cell cycle, DNA conformation change, DNA replication and repair mechanisms. Clusters 5,10, and 12 genes were associated with mRNA splicing via spliceosome, transport, protein localisation to nucleus and macromolecule, and primary and cellular metabolism, while cluster 7 genes were associated with ribosome biogenesis.

Cluster 9 genes mainly localised in the ECM (Ccl2, Cav1, Plau, Pf4, Itgb3, Timp1, Flt1) were enriched in wound response, ECM organisation, smooth muscle cell and myeloid leukocyte migration.

Taken together, our results show that administration of 4MU or siHAS2-LNP to mice with CCl_4_-induced fibrosis alleviated fibrosis by inhibiting fibroblast activation, cell proliferation, myeloid leukocyte and fibroblast migration. Furthermore, both antifibrotic agents muted inflammation and inhibited both transcription and translation machinery, which in turn diminished macromolecule biogenesis.

### Validation of RNAseq selected targets by qPCR

**Figure 13:**
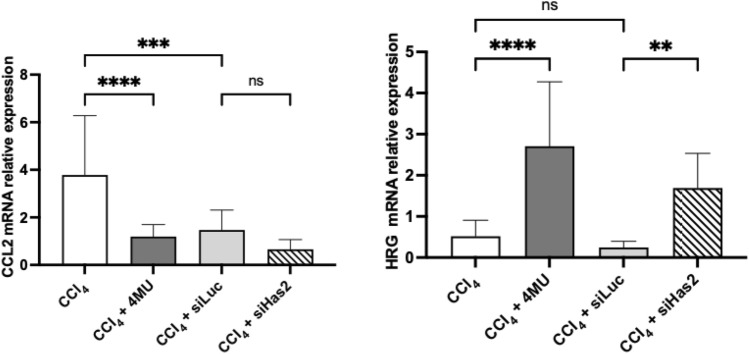
Relative mRNA expression of Ccl2 and Hrg. Data are presented as mean ± SD, n = 4 One-way ANOVA, ****p < 0.0001, ***—p < 0,001, **—p < 0,01, * -p < 0,05, ns- not significant.

Finally, we confirmed the expression of two target genes identified through our transcriptomic analysis. The mRNA levels of chemokine ligand 2 (Ccl2) and histidine-rich glycoprotein (Hrg) were analysed via qPCR. Consistent with RNA-seq results, treatment with 4MU and siHAS2 increased the mRNA expression of Hrg and decreased the mRNA expression of Ccl2 compared to the model group (Fig. 13).

## Discussion

Hepatic fibrosis is a manifestation of chronic damage to the liver. Despite it being one of the most characterised pathologies, an effective therapeutic strategy remains elusive. This study investigated the macro and micro protective effects of blocking HA production using 4MU and siHAS2-LNP. We compared liver transcriptomic signatures resulting from both approaches to elucidate common and unique biological pathways that could be responsible for 4MU hepatoprotective activity. Despite some nonspecific effects of siLuc-LNP, our analysis revealed specific effects of siHAS2-LNP and identified several critical pathways common to both 4MU and siHAS2-LNP treatment.

The interference of cationic lipid nanoparticles (LNP) with inflammatory responses has been observed in several studies. This phenomenon has been previously reported, wherein cationic LNP of the same composition as those used in this study can amplify pre-existing inflammation. The authors demonstrated that injection of nonimmunogenic RNA formulated in LNP (modmRNA-LNP) potentiated existing LPS-induced inflammation in mice as observed by higher induction of IL-6 and macrophage inflammatory protein 2 (Mip-2) in serum from LPS-treated mice injected with modmRNA-LNP compared to mice treated with LPS only. This effect was specific to the LNP, acting independent of mRNA cargo, a phenomenon the authors termed inflammation exacerbation (IE). It was further established that the IE phenomenon is a reaction to LNP, not to modmRNA, as empty LNP led to the same inflammation potentiation effect and cytokine surge^76^. IE phenomenon was associated with pro-inflammatory cytokine surge, mainly Mcp-1, also known as Ccl2 (a potent inducer of migration and activation of Natural killer cells, NK)^77^.

We postulate that the IE phenomenon explains the antifibrotic effects of siLuc-LNP. Recent publications provide strong support for our hypothesis. NK cells have been shown to suppress HSC differentiation and induce apoptosis. NK cells constitute a significant portion of human circulating lymphocytes, accounting for approximately 5–15%. In the liver, they make up half of intrahepatic lymphocytes. In mice, the percentage of NK cells in the liver’s non-parenchymal cells (NPCs) population is roughly 2–5%. However, this number can increase to around 10% in nonalcoholic steatosis (NASH)^78^. Specifically, NK cells display antifibrotic capability by the cytotoxic attack of early activated or senescent HSCs^79^ or by induction of HSC apoptosis^80^. NK cell–mediated cytotoxicity includes recognition (contact and adhesion), effector degranulation and lysis, and termination (detachment)^81^. Adoptive transfer of CD27^+^CD11b^+^ double-positive NK cells attenuated CCl_4_-induced liver fibrosis in mice^82^. Thus, CCl_4_ injury, together with positively charged LNP, enhanced the recruitment of leukocytes into the liver, resulting in reduced collagen deposition synonymous with fibrosis.

Total liver RNAseq analysis showed that CCl_4_-mediated hepatic damage induces DNA replication and mitotic division, which is accompanied by an increased activity of biological processes that are associated with fibrosis progression, such as ECM organisation, angiogenesis, inflammation, chemotaxis and cell trafficking. Downregulated transcripts were enriched in hepatocyte-specific pathways associated with homeostatic hepatic function, such as xenobiotic, lipid, bile and steroid metabolism, which indicates a reduction in the proportion of hepatocytes with progression of fibrosis. Accordingly, our deconvolved transcriptome showed an increased proportion of innate immune cells and fibroblasts but a decreased proportion of hepatocytes in the fibrosis model. Furthermore, markers of hepatic damage (ALT, AST) and HSC activation (α-SMA) were also elevated in the model but were downregulated upon treatment with either 4MU or siHAS2-LNP.

The histological analysis showed that 4MU inhibits excessive collagen and HA deposition, HSC and macrophage colocalisation, as well as HSC activation. The transcriptome analysis revealed that 4MU exerts its hepatoprotective effect by restoring the antioxidant capacity of hepatocytes and the lipid balance disrupted by CCl_4._ Furthermore, 4MU halts subsequent macromolecule biogenesis by interfering with RNA processing machinery, which ultimately inhibits DNA replication and mitotic division. Our results align with several studies alluding to the role played by dyslipidaemia in promoting fibrosis^49,83^. It also underpins the cytostatic effects of 4MU observed in vitro^63^. Furthermore, these findings, together with our previous observations^42^, clearly demonstrate that the antifibrotic effect of 4MU cannot be solely attributed to its antioxidant function.

Enrichment analysis and functional annotation of transcriptome from siHAS2-LNP treated mice revealed that down-regulated transcripts were involved in ECM assembly and collagen fibril organisation. Interestingly, the knockdown of HAS2 was accompanied by the downregulation of cell migration-inducing hyaluronidase (CEMIP). It has been shown that CEMIP regulates the depolymerisation of HA and its over-expression correlates with fibroblast activation^84^. Fibrosis related, transcripts such as α-SMA, Col1a1 and Col3a1, were also downregulated. Furthermore, GO BP revealed that TGF-β and semaphorin-plexin signalling pathways were downregulated. In situ, siHAS2 reduced the deposition of collagen (Fig. 1) and HA (Fig. 6). These findings clearly suggest that siHAS2 suppresses fibrosis by directly modifying the balance between polymerisation and depolymerisation of HA in ECM.

Both 4MU and siHAS2-LNP exert hepatoprotective effects through the inhibition of HA production. This prompted us to characterise converging signalling pathways and phenotypic alterations that could potentially be downstream of HA signalling. To achieve this, we compared and contrasted the transcriptomic signatures of experimental groups administered with 4MU and siHAS2. GO BP enrichment analysis suggested that most genes affected by 4MU and siHAS2-LNP treatment were associated with ECM organisation, macromolecule metabolism, ER stress response, autophagy, redox homeostasis, and immune response.

It was an anticipated finding of this study that the primary impact of 4MU treatment and HAS2 knockdown would be on the ECM and its non-fibrillar proteins. Hepatic fibrosis is synonymous with collagen deposition, and we found that 4MU and siHAS2-LNP reduced collagen accumulation and inhibited the mRNA and protein levels of α-SMA, the marker of the HSC activation. In addition, our transcriptomic analysis showed downregulation of Ccl2 and upregulation of Hrg upon treatment with 4MU and siHAS2-LNP, which was confirmed by qPCR (Fig. 13). Hrg and Ccl2 regulate ECM turnover and homeostasis. Ccl2, with its receptor CCR2, induces Ly6C^hi^ monocyte infiltration into the liver in response to damage. Moreover, Ccl2 has been shown to drive inflammation-associated angiogenesis, and its inhibition reduced monocyte recruitment into the liver, resulting in limited angiogenic activity of the injured livers^85^. Hrg regulates the ECM by acting as an adaptor molecule in several ECM-associated functions, such as cell adhesion and angiogenesis. Hrg has been shown to promote fibrosis due to its role in hepatic macrophage polarisation and inflammation; however, Hrg inhibits angiogenesis^86^. A proteomics study that used CCl_4_ and DDC models of fibrosis to assess the changes in hepatic ECM composition revealed that Hrg was upregulated in the DDC model^87,88^. Together, these findings demonstrate that 4MU treatment and HAS2 knockdown inhibit angiogenesis in hepatic fibrosis.

Both 4MU treatment and HAS2 knockdown repressed expression of genes involved in transcription, ribosome biogenesis and translation: this limited DNA replication, mitotic division, and proliferation. In recent years, researchers have acknowledged the significance of the ECM in regulating tissue-specific gene expression, subscribing to the idea that the ECM is an extension of a cell and is an active modulator of cellular function. This phenomenon is referred to as “Dynamic Reciprocity”^89,90^. It was suggested that the ECM influences gene expression through transmembrane receptors and components of the cytoskeleton. The cytoskeleton’s association with the nuclear matrix, in turn, affects mRNA processing and rates of transcription, whilst the cytoskeleton interaction with polyribosome determines mRNA stability. These cellular responses, in turn, affect ECM assembly and composition^87,91,92^. Here, we postulate that the obstruction of HA synthesis, an essential ECM component, induced ECM disequilibrium, which ultimately inhibited gene expression.

The functional annotation of enriched genes revealed that 4MU and HAS2 knockdown can suppress the ER stress response induced by CCl_4_ in mice with liver fibrosis. The ER stress response has been shown to activate pro-inflammatory transcription factors such as NF-κB and AP-1 through IRE1α-mediated induction of TRAF2 signalling^93^. In addition, the induction of ER stress response has been shown to alter the function and activation of fibroblasts^94^. Recently, it has been reported that ER stress response signalling mediates Col1 secretion by HSCs through XBP1-dependent induction of transport and Golgi organisation 1 (TANGO1), thus promoting liver fibrosis^95^. Sustained activation of ER stress response enhances TGF-β–mediated myofibroblast differentiation partially via IRE1α-mediated degradation of miR-150, a negative regulator of α-SMA expression^93,96^.

## Conclusion

Fibrogenesis in the liver is a multifaceted process involving the dynamic interaction of different cell types. Our hepatic transcriptome analysis revealed that the antifibrotic effect of 4MU is exerted through the modulation of multiple pathways, such as ECM organisation, cell proliferation, cell migration, ER stress response and HSC activation. These processes are critical for the initiation and progression of hepatic fibrosis. Our results provide further clues about the pharmacological targets of 4MU. Furthermore, we show that successful treatment of liver fibrosis may be achieved with small molecule HAS2 inhibitors, which underpins the viability of repurposing 4MU as an antifibrotic agent. Additionally, RNAi therapy using siHAS2 is a feasible and practical approach to ameliorating fibrosis. Our findings highlight the potential of 4MU and siHAS2 as promising agents for combating hepatic fibrosis and targeting its critical pathways.

## Material and methods

### Animal experiments

All animal procedures were conducted following the Russian Academy of Science Guidelines for Animal Experimentation and were approved by the Institute of Developmental Biology RAS Ethics committee (Protocol №60 from 16.06.2022). All animal experiments in this study were conducted in accordance with the ARRIVE guidelines for reporting experiments involving animals. All methods were carried out per relevant guidelines and regulations^97^. Eight-week-old female Balb/c mice purchased from the Scientific Center for Biomedical Technologies of the Federal Medical and Biological Agency, Moscow, Russia, weighing on average 18.22 g, were housed in a 12-h light/dark cycle with an ad libitum access to food water at a room temperature of 22 °C and relative humidity of 55%–65%. Mice were allowed to acclimate to the conditions for 2–3 days before the experiment.

### siRNA synthesis and titration

Eight siRNAs targeting mouse HAS2 gene sequence with the lowest off-target potential were designed. siRNAs were synthesised and chemically modified (methylated pyrimidine nucleotides (2′OMe) and phosphorothioate linkages) to stabilise siRNA in vivo, reducing the off-target potential of the sense strand and minimise the immune response (Supplementary Table S1). NIH 3T3 cells were transfected with siRNAs using Lipofectamine RNAiMAX (Invitrogen) to test the knockdown efficiency by qPCR for each siRNA. The siRNA with the highest potency and the lowest IC50 was selected for further experiments. The selected siRNA was formulated into lipid nanoparticles (LNPs). Briefly, the water phase contained siRNA duplex and ethanol phase with lipids (C12-200, 1,2-distearoyl-sn-glycero-3-phosphocholine (DSPC, Avanti Polar Lipids), cholesterol (Sigma), C14 PEG 2000 (Avanti Polar Lipids) at a 50:10:38.5:1.5 molar ratio) was mixed in a microfluidic PDMS chip. LNPs were dialysed overnight against PBS. LNP sizes were measured by dynamic light scattering (ZetaSizer, Malvern Instruments). The mean diameter of the particles prepared for injections was about 90–120 nm.

### siRNA injection and 4MU administration

4MU (Sigma-Aldrich, Cat # M1381) was administered at a dose of 600 mg/kg daily *per os* by gavage in a mixture with 0.5% carboxymethyl cellulose. Control mice were gavaged similarly with 0.5% carboxymethyl cellulose slurry. The administration of 4MU commenced two days prior to the first CCl_4_ IP injection. Nanoparticle encapsulated siRNA for HAS2 and luciferase was intravenously administered through tail vein injection. The initial siRNA dose was 1 mg/kg, and the subsequent doses were 0,5 mg/kg. The administration of nanoparticle-encapsulated siRNA also commenced two days prior to the first CCl_4_ IP injection and was done twice a week for two weeks. The control groups (without fibrosis) n = 3 and the experimental groups (with fibrosis) n = 8 per group. At the end of the experiment, animals were terminally anaesthetised with 5% isoflurane (5AGG9621, Baxter Corporation) and humanely sacrificed by rapid exsanguination followed by cervical dislocation. Liver samples were collected for RNA isolation and histological examination.

### RNAseq

#### RNA isolation and library preparation

PolyA mRNA was isolated from liver samples using Trizol. Illumina cDNA libraries were constructed using the NEBNext Ultra II Directional RNA Library Prep Kit for Illumina (New England BioLabs, MA, USA) following the manufacturer’s protocol. cDNA libraries were sequenced using the NextSeq500 instrument (Illumina, San Diego, CA, USA). Sequencing was carried out with a read length of at least 85 bp in the paired-end mode, with an average number of reads per sample not less than 30 million.

#### RNASeq data analysis

Raw reads were trimmed with Trimmomatic (v0.39) to remove the adapters. The trimmed reads were aligned to the Mus musculus genome (assembly GRCm39.105) using the HISAT2 algorithm. The following analysis was performed in R version 3.4.2. Gene read counts were calculated using the R subread package, function featureCounts (with parameters count MultiMap-pingReads = F, isPairedEnd = TRUE). Genes with less than 10 reads on average per sample were filtered out. Correlation analysis was conducted to check the data for self-consistency: MDS-based Spearman correlation and PCA using normalised gene counts as CPM (count per million). DEGs were identified using the DESeq2 R package. Gene ontology analysis of DEGs was performed using the ClusterProfiler package with all significant genes as background. Pathway enrichment analyses were done across Bioplanet 2019, GO Molecular function, MSigDB_Hellmark_2020 and KEGG_Pathways_2019 CHEA 2016^98–100^ databases to annotate differences in molecular functions, biological processes and signalling pathways between experimental groups with Enrichr^101^.

#### Cell type deconvolution of liver bulk RNASeq

Two single-cell reference data sets of mouse liver were mined from^102^. Processing, analysis, and visualisation were performed using Seurat R package v.4.3.0^103^. Cell type proportion predictions of bulk RNAseq data based on pseudo-bulk mixtures were performed using the granulator R package following the authors’ guidelines.

#### Protein‑protein interaction (PPI) networks

The DEGs were mapped using the SearchTool for the Retrieval of Interacting Genes/Proteins (STRING; http://string-db.org; version 10.0) online database, and protein‑protein interaction (PPI) networks were constructed. The PPI networks were exported to Cytoscape 3.7 for visualisation. The most significant modules were identified using the Molecular Complex Detection(MCODE) plug-in of Cytoscape. The selection criteria were: MCODE scores > 5; degree cut‑off = 2; node score cut‑off = 0.2; maxdepth = 100; and k‑score = 2. GO term and KEGG pathway enrichment were assessed for the functional analysis.

### Histology and immunofluorescence

#### Mallory Trichrome staining

Freshly isolated liver samples were cut into 3 × 3 mm pieces and fixed in 10% buffered formalin for 24 h. After an extensive wash in diH2O, samples were dehydrated in isopropanol solutions with rising concentration from 70 to 100%, followed by two immersions in xylene and then embedded in Histomix (Biovitrum, Russia) at 56 °C. Embedded tissue samples were sectioned by microtome at 5 µm slices and mounted onto SuperFrost glass slides. For Mallory staining, we used a commercially available kit (Biovitrum, Russia). Sections were analysed using a Keyence BZ9000 BioRevo microscope (Yokogawa Electric Corporation, Japan).

The amount of Mallory trichrome stained tissue area in tissue sections was quantified on at least 10 view fields per slide at 100× magnification using NIH ImageJ software.

#### Immunofluorescence

For immunofluorescence assessment (IF), we used separate groups of mice (n = 4–8) following the protocol previously described by^42^. Mice were terminally anaesthetised by 5% isoflurane (5AGG9621, Baxter Corporation) inhalation and perfused through their left ventricle with PBS, followed by 10% buffered formalin (Biovitrum, Russia) for 20 min. Liver lobes were cut off and additionally fixed in 10% buffered formalin for 2 h at RT after samples were washed by PBS and cut into 5 µm slices by vibratome sectioning.

Vibratome sections were permeabilised in PBS-0.2% Triton X-100 for 1 h, followed by incubation with primary antibody for two days at + 4 °C. After several washes with PBS-0.2% Triton X-100, sections were incubated with appropriate secondary antibody for one night. Washed sections were mounted in TDE-mounting media and analysed by laser scanning confocal microscopy. Images were taken by a Carl Zeiss LSM800 confocal microscope (Carl Zeiss, Germany) equipped with 25 × oil immersion objective (LCI “Plan-Neofluar” 25 × /0.8 Imm Corr Ph2 M27, Carl Zeiss), 488 nm, 561 nm and 640 nm wavelength diode-lasers were used for fluorochrome excitation. Mosaic frame Z-stack, 4 × 4 observation field in size, were scanned on each sample in multitrack mode with 2 um optical section step.

The following primary and secondary antibodies were used:

Primary Ab: CLEC4F (AF2784, R&D Systems, 1:300); Desmin (ab32362, Abcam, 1:300); α-SMA (701457, Invitrogen, 1;100).

Secondary Ab: Donkey anti-Goat Alexa Fluor® 555 (A-21432, Invitrogen, 1: 1000); Donkey anti-rabbit Alexa Fluor 488 (A-21206, Invitrogen, 1:1000); Wheat germ agglutinin (WGA) Alexa Fluor 680 conjugate (W32465, Thermo Fischer Scientific, 1;1000) Alexa Fluor 647 Tyramide SuperBoost kit Streptavidin (B40936, Invitrogen).

We utilised biotinylated hyaluronic acid binding protein (HABP) following the method described by^104^ to detect hyaluronic acid. HABP (385911-50UG, Millipore) was used at a dilution of 1:100. First, paraformaldehyde-fixed paraffin-embedded liver sections were dewaxed and rehydrated in xylene-alcohol, then rinsed in diH2O and PBS. As a negative control, bovine testis hyaluronidase treatment was used (100 ug/ml, 1 h, 37 °C). The Avidin–biotin blocking system (SP-2001, Vector Laboratories, Burlingame, CA, USA) was utilised to prevent nonspecific binding following the manufacturer’s instructions.HABP (385,911-50UG, Millipore, Burlington, MA, USA) in PBS-0.2% Triton X-100 was applied overnight and incubated at room temperature. Following a thorough wash with PBS, the HABP signal was detected using Streptevidin-Cy3 (S6402-1ML, Sigma-Aldrich Co, St Louis, MO, USA). Finally, the samples were mounted in Prolong Gold antifade mounting media (P36930, Thermo Fisher, Waltham, MA, USA).

#### Colocalisation assay

To estimate colocalisation between CLEC4F^+^ macrophages and Desmin^+^ HSCs, we choose Manders’ Colocalization coefficient (MCC)^42^. For paired markers R and G, two different values of MCC derived, M1, the fraction of R in compartments containing G and M2, the fraction of G in compartments containing R. M1 and M2 were calculated as:

$${\text{M1}} = \sum {\text{iRi coloc}}\sum {\text{iRi}}$$

$${\text{M2}} = \sum {\text{iGi coloc}}\sum {\text{iGi}}$$

The analysis was done using the colocalisation module of Imaris 7.4.2 software (Bitplane, Switzerland).

#### Serum collection and liver function tests

Whole blood sampled from the left ventricle was left to clot for 30 min at rtp. The clotted blood was centrifuged at 2000 g, 4 °C for 10 min and serum was extracted and then stored at − 20 °C. Liver marker enzymes (alanine aminotransferase (ALT), aspartate aminotransferase (AST), and total cholesterol were estimated using standard diagnostic kits at a commercial biomedical analytical laboratory (ChanceBio, Moscow, Russia).

### Cell culture

#### Hepatic stellate cell isolation

The protocol for isolating HSCs was performed according to the methodology described by^105^. Briefly, seven healthy male Balb/c mice, aged 20 weeks with an average weight of 24 g, were anaesthetised with isoflurane. The mice were then perfused through the portal vein with EGTA solution to eliminate residual blood and avoid contamination with red blood cells. Following this, the mice were perfused with Pronase E solution for 5 min at a flow rate of 6.5 ml/min, followed by Collagenase P perfusion for 7 min at the same flow rate. The liver was then excised from the diaphragm and surrounding organs, minced under sterile conditions, and transferred to a pre-warmed Pronase E-Collagenase P solution with 1 ml of DNase I. The liver suspension was further digested for 20 min at 37 °C on a shaking incubator. The liver suspension was then filtered through a 70 uM cell strainer and centrifuged for 10 min at 600 g and 4 °C. The supernatant was carefully aspirated, and the pellet was supplemented with DNase 1. The pellet was resuspended in GBSS solution, and the cell resuspensions were pooled into one 50 ml falcon tube. Subsequently, Nycodenz solution was added to the tube, and 10 ml of the cell suspension was transferred to a 12 ml-gradient centrifugation tube, resulting in 12 tubes. The gradients were centrifuged for 15 min at 1500 g at 4 °C without a brake. The interphase containing stellate cells was aspirated, supplemented with GBSS solution, and then centrifuged again for 10 min at 600 g and 4 °C. The supernatant was aspirated and discarded, and the pellet was resuspended in DMEM supplemented with 10% heat-inactivated FBS, 1% Penicillin/Streptomycin, 2 mM l-Glutamine, 1 mM sodium pyruvate, and 10 mM HEPES. The cells were seeded on a tissue culture flask and incubated at 37 °C and 5% CO_2_. 4 h later, after HSC had adhered to the surface, the media was refreshed to remove dead cells and debris.

#### Cell transfection

HSCs were seeded on a 96-well plate at a density of 2*10^4^ cells/well. The next day, HSCs were transfected with 10 nM siRNA final concentration following the Lipofectamine RNAi Max. 24 h post-transfection, TGF-β 20 ng/ml and 4MU (0.25 mM) were added. After 72 h, cells were fixed and stained for α-SMA, a marker of activated HSC.

#### qPCR

Total RNA was extracted using ExtractRNA (Evrogen, Russia), and the subsequent cDNA synthesis was performed using QuantiTect Reverse Transcription Kit (Qiagen, Germany). Quantitative RT-PCR was performed using an Applied Biosystems StepONE Plus Real-Time PCR System (Thermo Fisher Scientific, USA) and qPCR mix-HS SYBR master mix containing SYBR Green I dye (Evrogen, Russia). The mRNA levels of genes of interest were quantified via ΔΔCt Relative quantification method with GAPDH as a housekeeping reference target. Each reaction had three technical replicates, and only the sybr green master mix was used as a negative template control. Primer sequences are listed in Table 2 below.

**Table 2:** Oligonucleotide sequence of primers used for qPCR.

Gene name	Forward: 5’-3’	Reverse: 5’-3’
α-SMA	CTGAAGAGCATCCGACAC	AGCCTGAATAGCCACATACAT
HAS2	AGGTCGGTGTGAACGGATTTG	GAGAGCCTCAGGATAACT
Hyal1	AACAAGTACCAAGGAATCAT	GAGAGCCTCAGGATAACT
Col1a1	CCGCAAAGAGAGTCTACATGTC	CTGACTTCAGGGATGTCTTC
GAPDH	ACCTGCCAAGTATGATGA	GGAGTTGCTGTTGAAGTC
Col3a1	AACACGCAAGGCAATGAGAC	AAGCAAACAGGGGCCAATGTC
CCL2	CTCTTCCTCCACCACCAT	CTCTCCAGCCTACTCATTG
HRG	ACAGAAAGCAAGCCCTAAAG	GCAGAATCCAAAGACCAGAGG

### Statistical analysis

The findings of this study are presented using mean value ± standard deviation (SD) format, with all data points included in the analyses without any outliers being excluded from means or statistical significance calculations. To assess the significance of the previously calculated means, we conducted an ordinary one-way ANOVA, with a p-value of less than 0.05 considered statistically significant for all comparisons. We used the recommended multiple-comparison post hoc test in GraphPad Prism 9 to compare statistical differences between different populations.

### Institutional review board statement

The animal study protocol was approved by the Ethics Committee of the Koltzov Institute of Developmental Biology of the Russian Academy of Sciences (protocol number: 49; 22.07.2021).

### Supplementary Information

Supplementary Information.

## Data Availability

The datasets generated and analysed during the current study are available in the [Gene Expression Omnibus] repository, [accession number GSE241894].
