# Supplementary material for: Knockdown of Hyaluronan synthase 2 suppresses liver fibrosis in mice via induction of transcriptomic changes similar to 4MU treatment
Source: Sci Rep. 2024 Feb 2;14:2797. doi: 10.1038/s41598-024-53089-x (PMC10837461; doi:10.1038/s41598-024-53089-x)
Supplement: Supplementary file 1 — Supplementary Information. [file 41598_2024_53089_MOESM1_ESM.docx]

Supplementary Information

1. **siRNA screening and titration**

Table S1:HAS2 siRNA sequences with least off-target effect potential screened and titrated.

| Sequence number | Sense | Anti-sense |
| --- | --- | --- |
| Has2_1 | ccGAuuAAAuuGAAcAAAdTsdT | UUUUGUUcAAUUuAAUCGdTsdT |
| Has2_2 | cGuucAAAuGucAAAuuudTsdT | AAAAUUUGAcAUUUGAACdTsdT |
| Has2_3 | ccuucuAGGuGGuGuAAudTsdT | AAUuAcACcACCuAGAAGdTsdT |
| Has2_4 | cuGGAuGGcuuuuAAuAudTsdT | uAuAUuAAAAGCcAUCcAdTsdT |
| Has2_5 | uuAcGGAAAuGuuuGcAAdTsdT | AUUGcAAAcAUUUCCGuAdTsdT |
| Has2_6 | cuAuGuGGuucucAucAAdTsdT | AUUGAUGAGAACcAcAuAdTsdT |
| Has2_7 | cAuuGuGAGAGGuuucuAdTsdT | AuAGAAACCUCUcAcAAUdTsdT |
| Has2_8 | ccuuGuGGcGAAGcAAAAdTsdT | UUUUUGCUUCGCcAcAAGdTsdT |

A




B

Supplementary Figure S1: siRNA screening and titration. (A)NIH 3T3 cells were transfected with siHAS2, and qPCR was done to evaluate potency of knockdown of the 8 siRNA sequences designed). (B) si5 and si6 were selected for IC50 determination due to their knockdown efficiency and si5 IC50 was 52pM with a 95% CI range of 35 pM to 78 pM whilst that of si6 was 116pM with a 95% CI range 55pM to 249pM. si5 was used for in-vivo experiments.

1. **Effect of 4MU and RNA-LNP on serum triglycerides**

Supplementary Figure S2: Triglyceride levels in the serum of mice with CCl4 induced fibrosis treated with 4MU, siHAS2, siluc and 4MU/siHAS. Data are present as mean ± SD, n= 4. ^**^—P < 0.01,

1. **4MU reduce HAS2 expression and hyaluronan production in stimulated HSC.**

We assessed changes in gene expression in non-activated and TGF-b-activated HSCs. Three days of TGF-b stimulation led to a 100-fold increase in HAS2 expression. 4MU reduced the expression of HAS2 in all tested concentrations (0.1-0.5 mM). For all in vitro experiments, we used 0.25 mM 4MU. Consistent with the decrease in HAS2 expression, the HA level in cell media was also reduced to the control level by 4MU treatment. More importantly, we observed no adverse effects on cell viability following 4MU treatment. We observed a significant difference in HA inhibition between 0.1-0.5 mM of 4MU.

B

A

**
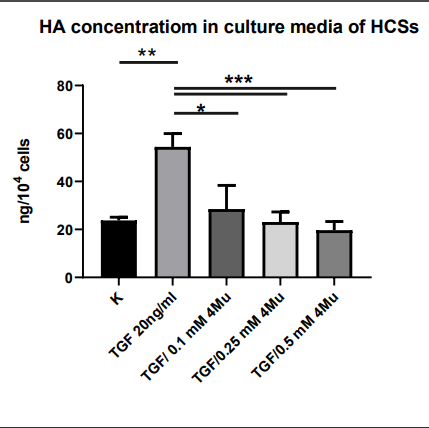
**

**
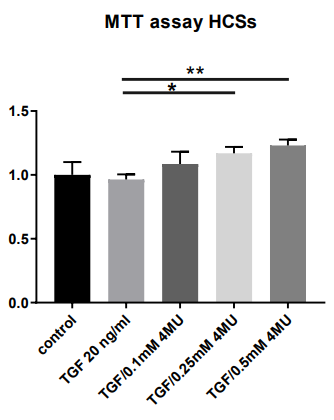
** **
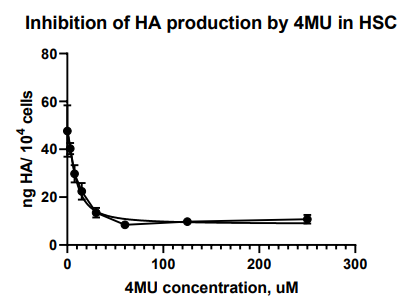
**

D

C

Supplementary Figure S3: 4MU at a concentration of 0.25 mM inhibits HAS2 mRNA expression and HA production in non-activated and TGF-b activated HSCs. (A) Relative mRNA expression analysis of HAS2 in non-activated and TGF-b activated HSCs. (B) HA concentration secreted into the media by non-activated and TGF-b activated HSCs treated with varying 4MU concentrations. 4MU inhibited HA secretion into media in a dose dependent fashion. (C) Determination of the cytotoxic activity of 4MU on non-activated and TGF-b activated HSCs using MTT assay. (D). 4MU dose response curve. IC50 dose was determined via titration in NIH3T3 cells. The titration dose ranged from 0-250uM; non-linear fit regression analysis revealed IC50 = 8.76 ± 1.12 uM. Data are presented as mean ± SD, n = 7 One-way ANOVA, ****p<0.0001, *** - p<0,001, ** - p <0,01, * -p<0,05, HSC=non activated HSC, aHSC=activated HSC

MTT assay

The MTT (Thiazolyl Blue Tetrazolium Bromide) used in the experiment was purchased from PanEco (Q105, Russia). The MTT was dissolved in PBS to a final concentration of 5 mg/mL. Next, 10 μL of the solution was added to 100 μL of cell culture medium and incubated for 3 hours under conditions of 37°C and 5% CO2. After the incubation period, the cell plates were centrifuged at 1500 rpm for 5 minutes. The media was then carefully removed, and 100 μL of pure DMSO was added to each well. Finally, the absorbance of the resulting solution was measured at 570 nm using a multi-plate reader.

1. **Transcriptomic analysis of DEGs**

Table S2:The top 20 upregulated and downregulated DEGs between non-treated and CCL_4_ treated mice.

| ENSEMBL | Gene Symbol | log_2_ FC | | padj |
| --- | --- | --- | --- | --- |
| ENSMUSG00000028312 | Smc2 | 9,78 | 3,39E-36 | |
| ENSMUSG00000024242 | Map4k3 | 7,63 | 1,02E-27 | |
| ENSMUSG00000042439 | Zfp532 | 7,41 | 2,43E-24 | |
| ENSMUSG00000063568 | Jazf1 | 7,28 | 3,92E-62 | |
| ENSMUSG00000056536 | Pign | 6,96 | 6,06E-25 | |
| ENSMUSG00000001555 | Fkbp10 | 6,69 | 7,16E-48 | |
| ENSMUSG00000001855 | Nup214 | 6,65 | 3,35E-24 | |
| ENSMUSG00000047804 | Akap10 | 6,43 | 1,57E-17 | |
| ENSMUSG00000037519 | Ppfia1 | 6,4 | 8,32E-15 | |
| ENSMUSG00000020607 | Lratd1 | 6,15 | 1,28E-27 | |
| ENSMUSG00000020681 | Ace | 6,05 | 9,35E-23 | |
| ENSMUSG00000038976 | Ppp1r9b | 5,83 | 1,57E-18 | |
| ENSMUSG00000079553 | Kifc1 | 5,83 | 5,71E-15 | |
| ENSMUSG00000022414 | Tab1 | 5,76 | 9,72E-63 | |
| ENSMUSG00000022385 | Gtse1 | 5,63 | 3,80E-14 | |
| ENSMUSG00000049288 | Lix1l | 5,55 | 2,74E-35 | |
| ENSMUSG00000031398 | Plxna3 | 5,55 | 4,99E-36 | |
| ENSMUSG00000044641 | Pard6b | 5,47 | 5,80E-20 | |
| ENSMUSG00000046167 | Gldn | 5,46 | 1,49E-74 | |
| ENSMUSG00000004677 | Myo9b | 5,43 | 2,30E-14 | |
| ENSMUSG00000075551 | Cyp3a41a | -9,61 | 1,00E-10 | |
| ENSMUSG00000075552 | Cyp3a41b | -9,24 | 3,04E-62 | |
| ENSMUSG00000038656 | Cyp3a16 | -9,12 | 2,50E-16 | |
| ENSMUSG00000078674 | Mup11 | -9,04 | 1,42E-21 | |
| ENSMUSG00000005089 | Slc1a2 | -8,69 | 0 | |
| ENSMUSG00000069668 | Sult3a1 | -7,53 | 8,95E-43 | |
| ENSMUSG00000092008 | Cyp2c69 | -6,27 | 1,71E-66 | |
| ENSMUSG00000078680 | Mup10 | -5,51 | 7,83E-13 | |
| ENSMUSG00000078686 | Mup9 | -5,46 | 6,89E-18 | |
| ENSMUSG00000074375 | Sult2a3 | -5,34 | 8,86E-48 | |
| ENSMUSG00000005547 | Cyp2a5 | -5,27 | 1,60E-13 | |
| ENSMUSG00000096688 | Mup17 | -5,05 | 4,64E-17 | |
| ENSMUSG00000038754 | Elovl3 | -4,94 | 1,85E-22 | |
| ENSMUSG00000089873 | Mup13 | -4,65 | 1,14E-10 | |
| ENSMUSG00000038370 | Pcp4l1 | -4,15 | 7,02E-79 | |
| ENSMUSG00000078688 | Mup2 | -4,14 | 4,26E-22 | |
| ENSMUSG00000089694 | Nat8f7 | -4,11 | 8,90E-25 | |
| ENSMUSG00000032808 | Cyp2c38 | -4,08 | 7,10E-72 | |
| ENSMUSG00000030934 | Oat | -4,02 | 4,40E-17 | |
| ENSMUSG00000026473 | Glul | -3,97 | 0 | |

Table S3: GO_BP terms ordered by FDR. Count: number of genes enriched in the corresponding pathway; FDR, false discovery rate.

| #term ID | Term description | Count | FDR |
| --- | --- | --- | --- |
| GO:0044281 | Small molecule metabolic process | 61/1450 | 1.28e-06 |
| GO:0006082 | Organic acid metabolic process | 41/821 | 1.08e-05 |
| GO:0019752 | Carboxylic acid metabolic process | 39/754 | 1.08e-05 |
| GO:0043436 | Oxoacid metabolic process | 40/800 | 1.08e-05 |
| GO:0006629 | Lipid metabolic process | 46/1032 | 1.26e-05 |
| GO:0032787 | Monocarboxylic acid metabolic process | 28/461 | 3.44e-05 |
| GO:0008202 | Steroid metabolic process | 18/217 | 0.00017 |
| GO:0008152 | Metabolic process | 170/7331 | 0.0010 |
| GO:0015850 | Organic hydroxy compound transport | 13/139 | 0.0024 |
| GO:0062012 | Regulation of small molecule metabolic process | 21/360 | 0.0025 |
| GO:0009056 | Catabolic process | 55/1680 | 0.0033 |
| GO:0044255 | Cellular lipid metabolic process | 33/797 | 0.0042 |
| GO:1901615 | Organic hydroxy compound metabolic process | 22/420 | 0.0056 |
| GO:0044237 | Cellular metabolic process | 149/6445 | 0.0069 |
| GO:0044238 | Primary metabolic process | 147/6369 | 0.0086 |
| GO:0071704 | Organic substance metabolic process | 155/6844 | 0.0094 |
| GO:0055088 | Lipid homeostasis | 12/145 | 0.0100 |
| GO:0016053 | Organic acid biosynthetic process | 15/240 | 0.0191 |
| GO:0008203 | Cholesterol metabolic process | 10/111 | 0.0240 |
| GO:0010565 | Regulation of cellular ketone metabolic process | 11/137 | 0.0252 |
| GO:0006869 | Lipid transport | 15/258 | 0.0352 |
| GO:0010876 | Lipid localization | 16/292 | 0.0352 |
| GO:0016042 | Lipid catabolic process | 15/260 | 0.0352 |
| GO:1901575 | Organic substance catabolic process | 45/1425 | 0.0352 |

Table S4: MCODE Cluster 2 genes GO BP terms ranked by FDR.

|  | Description | FDR | Count | Gene symbol |
| --- | --- | --- | --- | --- |
| GO:0034976 | Response to endoplasmic reticulum stress | 5.77E-15 | 11/216 | Calr, Hsp90b1, Dnajc3, Sdf2l1, P4hb, Dnajc10, Pdia3, Uggt1, Hyou1, Pdia4, Hspa5 |
| GO:0045454 | Cell redox homeostasis | 6.05E-7 | 5/44 | P4hb, Dnajc10, Pdia3, Pdia6, Pdia4 |
| GO:0035966 | Response to topologically incorrect protein | 8.04E-7 | 6/126 | Calr, Dnajc3, Sdf2l1, Uggt1, Hspa5, Manf |
| GO:0036503 | ERAD pathway | 7.84E-6 | 5/85 | Hsp90b1, Sdf2l1, Dnajc10, Uggt1, Hspa5 |
| GO:1905897 | Regulation of response to endoplasmic reticulum stress | 4.1E-4 | 4/82 | [Dnajc3, Hyou1, Hspa5, Manf] |
| GO:0034975 | Protein folding in endoplasmic reticulum | 0.0041 | 2/4 | Dnajc3, Dnajc10 |
| GO:0031204 | Posttranslational protein targeting to membrane, translocation | 0.0084 | 2/7 | Sec61a1, Hspa5 |
| GO:1903334 | Positive regulation of protein folding | 0.0084 | 2/7 | Pdia3, Pdia4 |
| GO:0071712 | ER-associated misfolded protein catabolic process | 0.0131 | 2/10 | Sdf2l1, Uggt1 |
| GO:0098761 | Cellular response to interleukin-7 | 0.0292 | 217 | P4hb, Pdia3 |
